# Metabolome profiling dissects the oat (*Avena sativa* L.) innate immune response to *Pseudomonas syringae* pathovars

**DOI:** 10.1371/journal.pone.0311226

**Published:** 2025-02-03

**Authors:** Chanel J. Pretorius, Paul A. Steenkamp, Ian A. Dubery

**Affiliations:** Department of Biochemistry, Research Centre for Plant Metabolomics, University of Johannesburg, Johannesburg, South Africa; Universitat Jaume 1, SPAIN

## Abstract

One of the most important characteristics of successful plant defence is the ability to rapidly identify potential threats in the surrounding environment. Plants rely on the perception of microbe-derived molecular pattern chemicals for this recognition, which initiates a number of induced defence reactions that ultimately increase plant resistance. The metabolome acts as a metabolic fingerprint of the biochemical activities of a biological system under particular conditions, and therefore provides a functional readout of the cellular mechanisms involved. Untargeted metabolomics was applied to decipher the biochemical processes related to defence responses of oat plants inoculated with pathovars of *Pseudomonas syringae* (pathogenic and non-pathogenic on oat) and thereby identify signatory markers that are involved in host or nonhost defence responses. The strains were *P*. *syringae* pv. *coronafaciens (Ps-c)*, *P*. *syringae* pv. *tabaci*, *P*. *syringae* pv. *tomato* DC3000 and the *hrcC* mutant of DC3000. At the seedling growth stage, metabolic alterations in the Dunnart oat cultivar (tolerant to *Ps-c*) in response to inoculation with the respective *P*. *syringae* pathovars were examined following perception and response assays. Following inoculation, plants were monitored for symptom development and harvested at 2-, 4- and 6 d.p.i. Methanolic leaf extracts were analysed by ultra-high-performance liquid chromatography (UHPLC) connected to high-definition mass spectrometry. Chemometric modelling and multivariate statistical analysis indicated time-related metabolic reconfigurations that point to host and nonhost interactions in response to bacterial inoculation/infection. Metabolic profiles derived from further multivariate data analyses revealed a range of metabolite classes involved in the respective defence responses, including fatty acids, amino acids, phenolic acids and phenolic amides, flavonoids, saponins, and alkaloids. The findings in this study allowed the elucidation of metabolic changes involved in oat defence responses to a range of pathovars of *P*. *syringae* and ultimately contribute to a more comprehensive view of the oat plant metabolism under biotic stress during host *vs* nonhost interactions.

## Introduction

Plants are sessile organisms that must adapt and survive recurring exposure events to a multitude of environmental stressors (abiotic and biotic). Biotic stress includes attacks by numerous pathogens like bacteria, fungi, nematodes, herbivores and oomycetes [1,2]. Plants have an elaborate and sophisticated detection and signalling system that allows them to recognise pathogen attacks quickly and initiate dynamic defence responses at a molecular level *via* a number of genes and transcription factors, including those from the phytohormonal pathway [3–5]. These interactions and how plants respond to various stress combinations has been widely studied and has led to the development of the ‘Stress Combinations and their Interactions in Plants Database’ (SCIPDb; http://www.nipgr.ac.in/scipdb.php) that provides data on the morpho-physio-biochemical (phenome) and molecular (transcriptome and metabolome) responses [6].

Plants utilise a multitude of pre-formed barriers such as the cell wall and cuticle to ward off potential pathogens. To counteract plant defence mechanisms, phytopathogens have evolved specialised mechanisms to infiltrate and overthrow host plant defences. In response, plants readily produce antimicrobial compounds that aid in preventing pathogen entry, maintaining cellular integrity and provide additional structural support. Pathogens that overcome these barriers trigger signalling pathways in the potential host that lead to the expression of defence response genes. Plants therefore rely on receptors to recognise pathogen invasion and activate signal transduction pathways that involve a multitude of genes and their products [7–10]. These signalling pathways activate calcium-dependent protein kinases, MAPKs, transcription factors, G-proteins, ubiquitin, and hormones that leads to various responses such as the hypersensitive response (HR), cell wall modification, stomatal closure, production of reactive oxygen species (ROS), specific proteins targeted at the invader (e.g., ß-glucananses and chitinases, defensins and protease inhibitors), or specific metabolites (e.g., phytoalexins) to protect against further infection [9,11,12].

The *Pseudomonas syringae* (*Ps*) species complex contains some of the best studied plant pathogens, existing as more than 60 pathovars able to infect a wide range of plants [13–15]. *P*. *syringae* utilises two types of secretion systems when infecting plants namely, the type II secretion system (T2SS) to transport proteins and the type III secretion system (T3SS) to deliver effectors into plant cells along with toxins that play an essential role in causing disease [16,17]. Numerous phytotoxins have been discovered in *Ps* strains, including coronatine, syringolin A, syringomycin, syringopeptin, phaseolotoxin, and tabtoxin. Coronatine mimics jasmonoyl-isoleucine (JA-Ile) and is one of the most well-studied *Ps* toxins [18,19]. Coronatine inhibits the activity of salicylic acid during stomatal closure, causing the stomata to reopen and allow pathogen invasion [20]. Once effectors are delivered and recognised by resistance (R) proteins, effector-triggered immunity (ETI) is triggered, and an HR induced [20] (S1 Fig). According to this scenario, effectors are ‘pathogen-derived Avr proteins that trigger resistance *via* activation of specific cognate host R proteins’. The latter are defined as ‘proteins that confers resistance by mediating direct or indirect recognition of a pathogen Avr protein’ [21]. Nucleotide-binding domain and leucine-rich repeat-containing proteins (NLRs) are ubiquitous in plants and a constitute a major class of immune receptors. NLRs detect specific pathogen effectors through diverse mechanisms and different models have been proposed to describe the mechanism of detection. These include the direct -, guard -, decoy -, integrated decoy—and NLR-like models [21,22].

Successful pathogens are able to overcome host plant defences by releasing/injecting effectors, however these are insufficiently potent in nonhost plant species. Effectors interfere with signal perception and transduction events within the host cell and may target transcription of specific defence-related genes [23,24]. Very little is known about the genes that control whether effectors can inhibit basal defence or not, despite significant progress in the understanding of the molecular features of nonhost resistance to plant diseases. Effectors from virulent pathogens are successful due to their ability to overthrow basal resistance compared to nonhosts. According to recent research, nonhost resistance should be regarded as a form of basal resistance that is polygenetically inherited and shares a high resemblance and correlation with innate plant resistance to adaptive pathogens [23,25]. Nonhost resistance can also be divided into type I and type II, based on the presence or absence of visual phenotypic changes. While type II nonhost resistance is linked to visual necrosis/cell death brought on by a HR, type I nonhost resistance does not cause any visual symptoms. In response to generic pathogen-derived elicitors like PAMPs, type I nonhost resistance often utilises passive or preformed barriers to activate defence mechanisms. Type I nonhost resistance is comparable to pattern-triggered immunity (PTI) and type II nonhost resistance to ETI. Despite some similarities between host and nonhost resistance, nonhost resistance is more complicated, and the mechanism of resistance might change depending on the pathogen and the plant species [26,27]. Phytoanticipins also play an important role in preventing infections by nonadapted pathogens. The most well-known examples of these preformed chemical defences are glucosinolates in Arabidopsis and in oat, the avenacins [28,29].

In view of the above, alterations in plant metabolism therefore have a significant impact on the fate of attempted infections. Accordingly, studying plant-microbe interactions such as those between healthy and infected or resistant and susceptible plants can reveal metabolomic changes in signalling pathways that are crucial for elucidating and determining the outcome of these interactions [30]. Since metabolites directly reflect biochemical processes (coordinated by genes, mRNA transcripts and proteins) the examination, at both a qualitative and quantitative level, provides new understanding of the biochemical systems behind the phenotype while also creating a thorough metabolic profile of the specific system [31,32]. Metabolites play a variety of roles in plant-pathogen interactions, including pathogen surveillance, signal transduction, enzyme control, cell-to-cell transmission, and antimicrobial activity [30].

Plants produce primary and secondary (or specialised) metabolites with most of the metabolome consisting of primary metabolites that are crucial for plant growth and development. Secondary metabolites, on the other hand, promote interaction with the environment and aids in plant survival under threatening conditions such as exposure to biotic and abiotic stressors. The synthesis of these specialised metabolites requires precursors that branch off from primary metabolite pathways [33,34]. The shikimate pathway is the first step in the biosynthesis of aromatic amino acids; when activated due to stress it produces tryptophan, tyrosine, and phenylalanine, which supports and boosts secondary metabolite production. Depending on the stress and environment, different metabolites accumulate in distinct plant organs. Phytoalexins, for example, exhibit antibacterial properties against phytopathogens and accumulate in high concentrations in leaves. In addition to their antibacterial effects, several of these metabolites help to build polymeric barriers against disease penetration [3,5]

Although *Ps* has been widely studied, limited studies have been done on oat under biotic stress. Here, these model *Ps* bacteria were used to study host and nonhost defence mechanism as well as the PTI and ETI triggered by the respective pathovars. An untargeted metabolomics approach was therefore applied to qualitatively profile and identify as many metabolites involved in the *P*. *syringae–Avena sativa* L. interaction as possible. Monitoring metabolite levels and changes therein, can complement and corroborate transcriptome and/or proteomic data on plant-pathogen interactions, thus revealing pathogen attack mechanisms. Transcriptomic and proteomic analyses are used to investigate changes in messenger RNA and proteins, respectively. Metabolites can also influence the outcomes of these gene-centered pathways. Thus, combining metabolomics and other omics data adds new layers of information to studies of plant-pathogen interactions, such as identifying metabolites with antimicrobial properties, metabolomic profile differences between infected and non-infected plants, and pathogenic requirements for infection and colonisation [30,35]. Therefore, the integration of other omics approaches or a multi-omics approach with metabolomics plays an important role in untangling the mechanisms underlying pathogen attacks [36].

In this report, an untargeted metabolomics approach was used to study the underlying mechanisms of *A*. *sativa* L. treated with various pathovars of *Ps*. While some oat cultivars exhibit a higher level of disease resistance/tolerance, the molecular mechanisms underlying these interactions are still poorly understood [37]. In addition to *P*. *syringae* pv. *coronafaciens* (pathogenic on oat), *P*. *syringae* pv. *tabaci* and *P*. *syringae* pv. *tomato* DC3000 were chosen as putative nonhost pathogens and the *hrcC* mutant of *P*. *syringae* pv. *tomato* DC3000 (lacking the T3SS) was chosen to represent only the PTI component of the oat response to infiltration with the *Ps* pathovars. A few studies have reported halo blight occurrence (caused by *Ps-c*), highlighting the economic and crop loss implications caused by the pathogen across several geographically dispersed countries [38–40]. By analysing the cellular and molecular responses between the plant and pathogen, sustainable means of combating disease could be developed and be particularly useful in breeding programs by identifying biomarkers and pathways associated with susceptibility, tolerance or resistance [41].

## Materials and methods

### Oat plant cultivation

Seeds of the Dunnart oat cultivar was procured (Agricol, Pretoria, South Africa) and chosen for further infection studies using an initial screening procedure, as described below. Seedlings were cultivated in 10 cm, 250 mL pots (±15 seeds per pot) with a pasteurized (90˚C) germination mixture soil (Culterra, Muldersdrift, South Africa) and watered twice weekly with a water-soluble chemical fertiliser (Multisol ‘N’, Culterra, Muldersdrift, South Africa). The were cultivated in greenhouse conditions with a 12 h/12 h (light/dark) cycle, a light intensity of approximately 80 μmol/m^2^/s, and a temperature of 25°C. The study was designed to track the cultivar’s response to bacterial infection over time (2–6 days post inoculation—d.p.i). Triplicate pots were grown for each time point and were cultivated under identical conditions. When the seedlings achieved 3-week maturity (three-leaf seedling stage), they were thinned out and selected for uniformity in developmental stage before treatment with the bacterial suspensions described in the following sections. The experimental design included three independent biological replicates.

### Preparation of *Pseudomonas syringae* pathovars

Dr. W. Kriel (Starke Ayres Seeds, Bredell, South Africa) provided an isolate pathogenic on oat namely *P*. *syringae*, pv. *coronafaciens* (*Ps-c*) with sequence information available on GenBank (PP940110), *P*. *syringae* pv. *tabaci* (*Ps-t*) was obtained from Prof. Y. Ichinose, Okayama University, Japan, and *P*. *syringae* pv. *tomato* DC3000 and its *hrcC* mutant from Dr. B. Kemmerling, University of Tuebingen, Germany. Inqaba Biotechnical Industries (Pretoria, South Africa) performed 16S rRNA sequencing to validate the pathovar identities. *Ps* isolates were then cultured and maintained on nutrient agar (Merck, Modderfontein, South Africa). A colony was selected under sterile circumstances in a laminar flow cabinet and cultivated overnight at 28°C in nutrient broth (Merck, Modderfontein, South Africa) on an orbital shaking incubator. The overnight culture’s OD_600_ was measured and diluted with 0.1% Tween 20 and phosphate buffered saline (PBS) to a value of around 0.3. The same dilution was used for non-inoculated nutrient broth as a control (applied to vehicle control plants). The pathogenicity of *P*. *syringae* strains on several oat cultivars was examined by drop-inoculation [42] on leaf segments (OD600 ≈0.1, 0.2, 0.3). The inoculated leaf segments were stored in a high-humidity container for 5 days in a regulated growth environment with a 12-hour light-dark cycle at 25°C and visual symptoms scored daily. ‘Dunnart’ was chosen for further metabolomic analysis as a cultivar with a tolerant reaction based on initial visual observation tests that demonstrated its ability to tolerate infection during the 5-day period.

### Inoculation of oat seedlings

At the three-leaf growth stage (approximately 21 days post emergence), the plants were treated by spraying with the *Ps-c*, *Ps-t*, DC3000 and *hrcC*^*−*^ bacterial suspensions (prepared in PBS with 0.1% Tween 20), diluted to OD_600_ ≈0.3. The vehicle control (VC) plants were sprayed with a solution free of the bacteria and the healthy control (HC) groups were untreated (i.e., not sprayed with either solution), all grown under normal growth conditions. The 50 mL of either the inoculum (containing the bacteria) or the control (0.1% Tween 20 in PBS) solution was evenly sprayed onto the leaves of the treated and VC groups respectively. The plants were then incubated in darkness in an incubator for 1 h to provide 100% relative humidity. Following the 1 h incubation, the plants were removed and another 50 mL of either inoculum or control solution was applied to the treated and VC groups, respectively, and further incubated for 6 h. After incubation, the plants were then exposed again to the same initial conditions: with cycles of 12 h light/dark, a light intensity of 80 μmol/m^2^/s and temperature of 25°C. Post-treatment harvesting of plants was done for treated, VC and HC groups at 2, 4 and 6 d.p.i. by cutting the leaves and immediately snap freezing with liquid nitrogen to quench metabolic activity associated with possible wounding and handling of the tissue. Leaves were stored at −80°C until metabolite extraction.

### Luminol-based reactive oxygen species (ROS) assay

ROS production was measured using a modified version of the luminometry method [43,44]. Briefly, leaves were pressure infiltrated with the respective bacterial suspensions (OD_600_≈0.3) or with H_2_O (control). The infiltrated sites were then excised and washed for 1 h in sterile water with agitation. The single leaf discs (4 mm in diameter) were then rinsed 3 times and individually placed into a 96-well microtitre plate with 20 μg/mL horseradish peroxidase (HRP) and 34 μg/mL of luminol (Sigma-Aldrich, St. Louis, MO, USA). The luminescence was then measured over a period of 30 min using a Synergy HT Biotek microplate reader (Biotek Instruments, Winooski, VT, USA).

### Peroxidase (POX) assay

POX activity associated with the innate immune response was measured using a modified version of the method described by [45]. Leaf discs (4 mm in diameter) were punched from leaves of the oat plants and washed with agitation for 1 h in 1 mL of a 1 x MS (Murashige and Skoog) salt solution to remove any background activity caused by cutting the leaves. The discs were then carefully transferred to individual wells in a 96-well plate using a small spatula to minimise damage. Each well then received 50 μL of 1 x MS solution with either H_2_O (control) or one of the respective overnight bacterial suspensions (OD_600_ ≈0.3). The plates were then sealed with parafilm and incubated for 20 h with agitation at room temperature. The leaf discs were removed and 50 μL of 5-aminosalicylic acid (Sigma-Aldrich, St. Louis, MO, USA) (1 mg/mL) at pH 6.0 with 0.01% hydrogen peroxide (Barrs Pharmaceuticals, Cape Town, RSA) was added to each well using a multichannel pipette in order to minimise timing differences. The reaction was allowed to proceed for 3 min and then stopped by adding 20 μM NaOH with a multichannel pipette in the same order as before, again to minimise timing differences and allow equal time for the enzymatic reactions to proceed. The plates were then analysed using a Synergy HT Biotek microplate reader (Biotek Instruments, Winooski, VT, USA) in absorbance mode at 600 nm (A_600_ nm).

### Metabolite extraction and sample preparation

The harvested leaf material was quenched with liquid nitrogen before being crushed into powder with a pre-cooled mortar and pestle. The samples were weighed (1 g) into 50 mL Falcon tubes with addition of 80% cold (4°C) aqueous analytical grade methanol (Romil Chemistry, Cambridge, UK).at a 1:10 m/v ratio. The suspensions were then homogenised with a probe sonicator (Bandelin Sonopuls, Berlin, Germany) at 55% power for 10 sec per sample. To avoid cross-contamination, equipment was cleansed between each sample. The homogenates were centrifuged at 5100 x *g* for 20 min at 4°C in a benchtop centrifuge after which the supernatants were kept and concentrated by evaporating the methanol under vacuum to approximately 1 mL using a rotary evaporator set to 55°C. The concentrated samples were transferred to 2 mL microcentrifuge tubes and dried in a centrifugal evaporator under vacuum. The dried extracts were then reconstituted by dissolving in 500 μL of 50% aqueous methanol (MilliQ deionised water and LC-grade methanol (Romil, Cambridge, UK). The samples were subsequently filtered through nylon syringe filters (0.22 μm) into chromatography vials fitted with 500 μL inserts, capped, and kept at 4°C until analysis.

### Sample analyses using ultra-high-performance liquid chromatography (UHPLC) and quadrupole time-of-flight mass spectrometry (qTOF-MS)

An Acquity UHPLC system (Waters Corporation, Manchester, UK) was used to analyse 2 μL of each sample, separated into its respective components using a binary solvent on an HSS T3 reverse-phase column (Waters Corporation, Billerica, MA, USA; 2.1 × 150 mm × 1.8 μm), thermostatted at 60°C. The solvents used were MilliQ water and acetonitrile (Romil Chemistry, Cambridge, UK), both containing 0.1% formic acid (Sigma-Aldrich, Munich, Germany) and 2.5% isopropanol (IPA, Romil, Cambridge, UK). The run was set to 30 min per injection with an elution gradient carried out *via* a binary solvent system consisting of 0.1% aqueous formic acid with 2.5% isopropanol (solvent A) and 0.1% formic acid and 2.5% isopropanol in acetonitrile (Romil, Cambridge, UK; solvent B) at a flow rate of 0.4 mL/min. The initial conditions were 95% A and 5% B and held for 1 min. A gradient was applied to change the chromatographic conditions to 10% A and 90% B at 25 min; and changed to 5% A and 95% B at 25.10 min. These conditions were held for 2 min and then changed to the initial conditions at 28 min. The analytical column was allowed to equilibrate for 2 min before each subsequent injection. Pooled quality control (QC) samples were also prepared to monitor the stability of the LC-MS system and assess the reliability and reproducibility of each analysis [46]. Additionally, blank samples (50% MeOH) were also randomly included in the run to monitor the background noise and potential carry-over of analytes. Each sample was analysed in triplicate (analytical/technical replicates), and together with the three biological replicates, this generated n = 9, to have the minimum required number of replicates for metabolomic studies that involve multivariate analyses.

A high definition SYNAPT G1 quadrupole time-of-flight (qTOF) mass spectrometry system (Waters Corporation, Manchester, UK) was coupled to the UHPLC chromatography system to detect metabolites and acquire data in both positive and negative electrospray ionisation (ESI) operation modes. The controlling software was MassLynx XS^TM^ (Waters, Manchester, UK). A reference calibrant, leucine encephalin (554.2615 Da) was used as the ‘lockmass’ calibrant and allowed for typical mass accuracies between 1 to 3 mDa. The respective capillary and sampling cone voltages were set as 2.5 kV and 30 V. The desolvation temperature used was 450°C, with the source temperature set to 120°C, cone gas flow was set to 50 L/h, and the desolvation gas flow set to 550 L/h. An *m/z* range of 50–1200 was set with a scan time of 0.1 s. The desolvation-, collision- and cone gas used at a flow rate of 700 L/h was high-purity nitrogen. Data was acquired using five different collision energies (MS^E^), ramping from 0–50 eV to cause fragmentation of the initial ions to ensure that information regarding the fragmentation of the respective compounds could be obtained for downstream structural elucidation and metabolite annotation [47,48].

### Data analysis

The data sets obtained were explored and processed using the applications manager, MarkerLynx XS^TM^ (Waters Corporation, Manchester, UK). The software makes use of a patented ApexTrack algorithm. The following parameters were used for processing: 2–23 min retention time (Rt) range and *m/z* range 50–1200 Da. The Rt window was set to 0.20 min and the mass window to 0.05 Da. The mass tolerance was 0.05 Da and the intensity threshold was set to 100 counts. The corrected data matrices were exported to ‘soft independent modelling of class analogy’ (SIMCA-version 16) software (Sartorius, Umeå, Sweden) for multivariate data analysis (MVDA). Unsupervised models, namely principal component analysis (PCA) and hierarchical clustering analysis (HCA) were used to reduce the dimensionality of the data sets and to explore the underlying structures and characteristics of the data. Supervised orthogonal projection to latent structures discriminant analysis (OPLS-DA) was used to compare the response of the oat plants to the different pathovars and identify discriminatory ions among the respective treatments. The OPLS-DA models were validated using rigorous validation methods that included cross-validated analysis of variance (CV-ANOVA) and receiver operator characteristic (ROC) analysis [31,49,50].

### Metabolite annotation and semi-quantitative comparison

Metabolites were identified based on their respective accurate masses, fragmentation patterns, and possible elemental compositions generated from the MarkerLynx^TM^ software. Each putatively suggested empirical formula was exported and searched for in various databases such as MetaCyc (https://metacyc.org/), Plant metabolic network (PMN) (https://plantcyc.org/), ChemSpider, Mass bank of North America (https://mona.fiehnlab.ucdavis.edu/), Dictionary of Natural Products (www.dnp.chemnetbase.com), and the Kyoto Encyclopedia of Genes and Genomes (KEGG, www.genome.jp/kegg/). Metabolites were putatively identified to level 2 of the Metabolomics Standards Initiative (MSI) [51] unless specified otherwise. The generated matrixes of the annotated metabolites were exported from MarkerLynx XS™ as.csv files. MetaboAnalyst 5.0 (https://www.metaboanalyst.ca/), an online platform for statistical, functional and integrative analysis of metabolomics data [52], was then utilised for visualisation of the MS files which contained the *m/z*, Rt and peak intensities of metabolites separated by chromatography and detected as ions by mass spectrometry. Data processing, integrity, missing values, filtering and normalisation were performed on MetaboAnalyst 5.0 followed by Pareto-scaling before statistical analyses to reduce variance within the features. A comparison of the presence and relative concentration/intensity of the identified metabolites among the various treatments were performed *via* dendrogram heatmap analyses using a Pearson distance measure and the Ward clustering algorithm in MetaboAnalyst 5.0 to visualise the distribution of metabolites. Additionally, the software’s metabolomics pathway analysis (MetPA) tool was employed to identify the crucial metabolic pathways induced in oat treated with the respective *P*. *syringae* pathovars. Each annotated metabolite’s KEGG (Kyoto Encyclopedia of Genes and Genomes; www.genome.jp/kegg/pathway.html) identifier was employed as an input, and the KEGG metabolic pathways (*Arabidopsis thaliana*) served as the knowledge base for the construction of the pathways.

## Results

### Evaluation of the oat leaf symptoms in response to the *Pseudomonas syringae* pathovars

**Fig 1:**
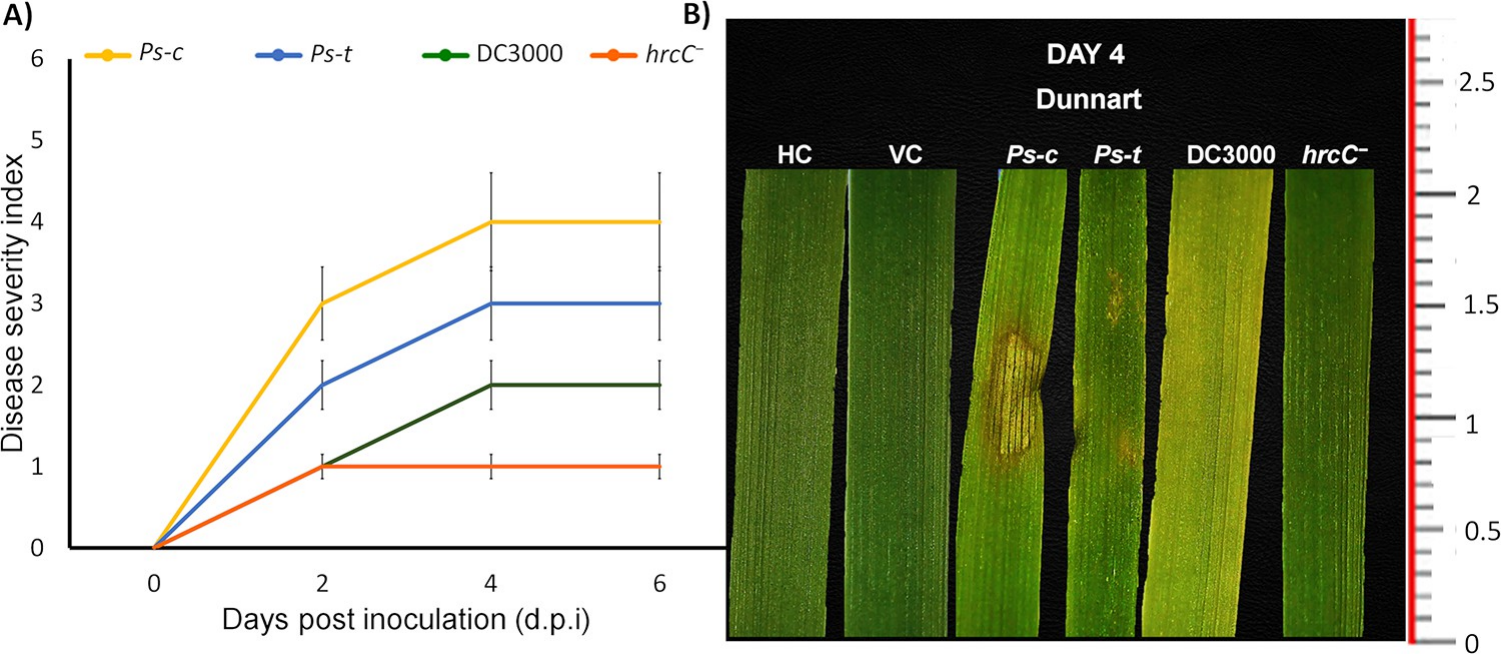
Disease severity rating and of phenotypical symptom development of the Dunnart oat cultivar after inoculation with *Ps-c*, *Ps-t*, DC3000 and *hrcC* mutant. **(A)** Disease severity range from 0 = Symptomless (no visual symptoms are observed), 1 = Limited amount of lesions or yellowing are present (5% of leaf material start presenting visual symptoms), 2 = Lesions and yellowing spread over the surface of the leaves (6%-11% of leaf material present visual symptoms), 3 = Lesions are present in moderate amounts (11%-20% of leaf material present visual symptoms), 4 = Lesions are present in severe quantities (21%-50% of leaf material present visual symptoms), 5 = Limited leaf wilting occurs (6%-11% of leaf material are yellow and wilted). Error bars indicate the standard deviation. **(B)** At 4 d.p.i. Dunnart showed typical halo blight symptoms upon infection with *Ps-c* such as the water-soaked lesion and the development of a characteristic yellow halo, small brown necrotic spots were apparent upon infection with *Ps-t* and typical chlorosis of the leaf upon infection with DC3000, however no visible symptoms were noted for the *hrcC*^*−*^ treatment.

The disease severity of the oat cultivar (Dunnart) to the respective pathovars was assessed using visual observation, with scoring ending 6 d after first infection. Visual observation scoring was used with a scale of 0 to 5, with 0 indicating no symptoms, 1–3 indicating minor symptoms, 3–5 indicating moderate to severe disease symptoms (Fig 1A). The typical symptoms associated with *Ps-c* infections include a HR that is characterised by necrosis at the inoculation site where a water-soaked lesion presented followed by the appearance of a characteristic yellow halo (Fig 1B). Typically, *Ps-t* causes brown spots that spreads across the surface of the leaf and later develops yellow halos, however when constrained by veins, the spots turn angular. Tabtoxin is produced by both of these pathovars and is responsible for the characteristic yellow halo formation. DC3000 infection is associated with chlorosis of the leaves, as can be seen in Fig 1B, which is mainly due to the presence of the phytotoxin coronatine (COR). The *hrcC* mutant lacks the T3SS, therefore its growth in the plant is restricted. Some studies have found that tomato plants inoculated with *hrcC*^*−*^ still developed some chlorotic patches that are suggestive of COR formation, however there was a lack of necrotic lesions that the wild type (DC3000) typically produces [53].

### Perception assays

#### ROS luminescence assay

**Fig 2:**
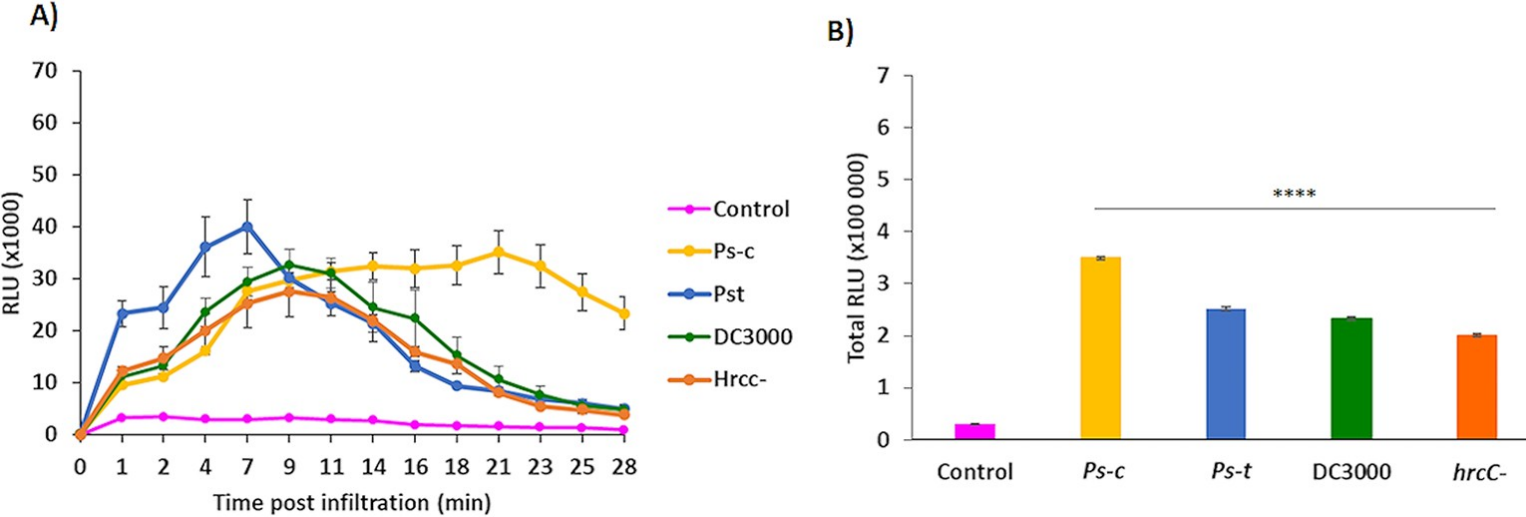
Chemiluminescence determination of reactive oxygen species generation by leaf disks from oat seedlings in response to various *P*. *syringae* treatments. The pathogen interaction and responses are visualised using the luminescence assay that monitors the kinetics of ROS production over time. **(A)** The rate of ROS production over a period of 0–28 min in response to the respective bacterial treatments. The light released owing due to the oxidation of luminol is represented as relative light units (RLU). The average values ± standard error of the mean (SEM) for each time point on the curve are shown (*n* = 36). **(B)** The sum of the integrated area under the curve shows the overall ROS production for the Dunnart cv as relative luminescence units (RLU) over 28 min as a result of the respective treatments (*Ps-c*, *Ps-t*, DC3000 and *hrcC* mutant). A paired Student’s t-test was used to compare the treatments to the control (**** = *p* < 0.0001).

The amount of light released owing to the oxidation of luminol by HRP is measured and represented in relative light units (RLU) to determine the ROS burst. The data provided is portrayed as the amount of RLU generated every minute over a 28 min period to track ROS production as an early defence response (Fig 2). Data is also represented as total RLU generated over the same period. The luminol based perception assays elucidates the capacity of the plants to detect and respond to infection with the respective *Ps* pathovars. The luminescence curves and total RLU indicate the production of ROS by the oat leaf discs in response to exposure with *Ps-c*, *Ps-t*, DC3000 and *hrcC*^*−*^ compared to the control (dH_2_O). Twelve independent experiments were carried out with *n* = 36 samples (3 biological replicates for each treatment condition). The total RLU graphs for the respective treatments show that the *Ps-c* treatment produced the greatest response followed by *Ps-t*, DC3000 and the lowest production seen in the *hrcC*^*−*^ treatment. The ROS burst for the *Ps-c* treatment lasted approximately 23 min before gradually decreasing back to basal levels. Treatment with *Ps-t* reached a maximum at 7 min and lasted for about 21 min. DC3000 treatment reached a maximum between 7–9 min and returned to basal levels at around 21 min post-treatment. The *hrcC*^*−*^ treated group showed a shorter burst with lower RLU compared to the other treatments.

#### POX activity

**Fig 3:**
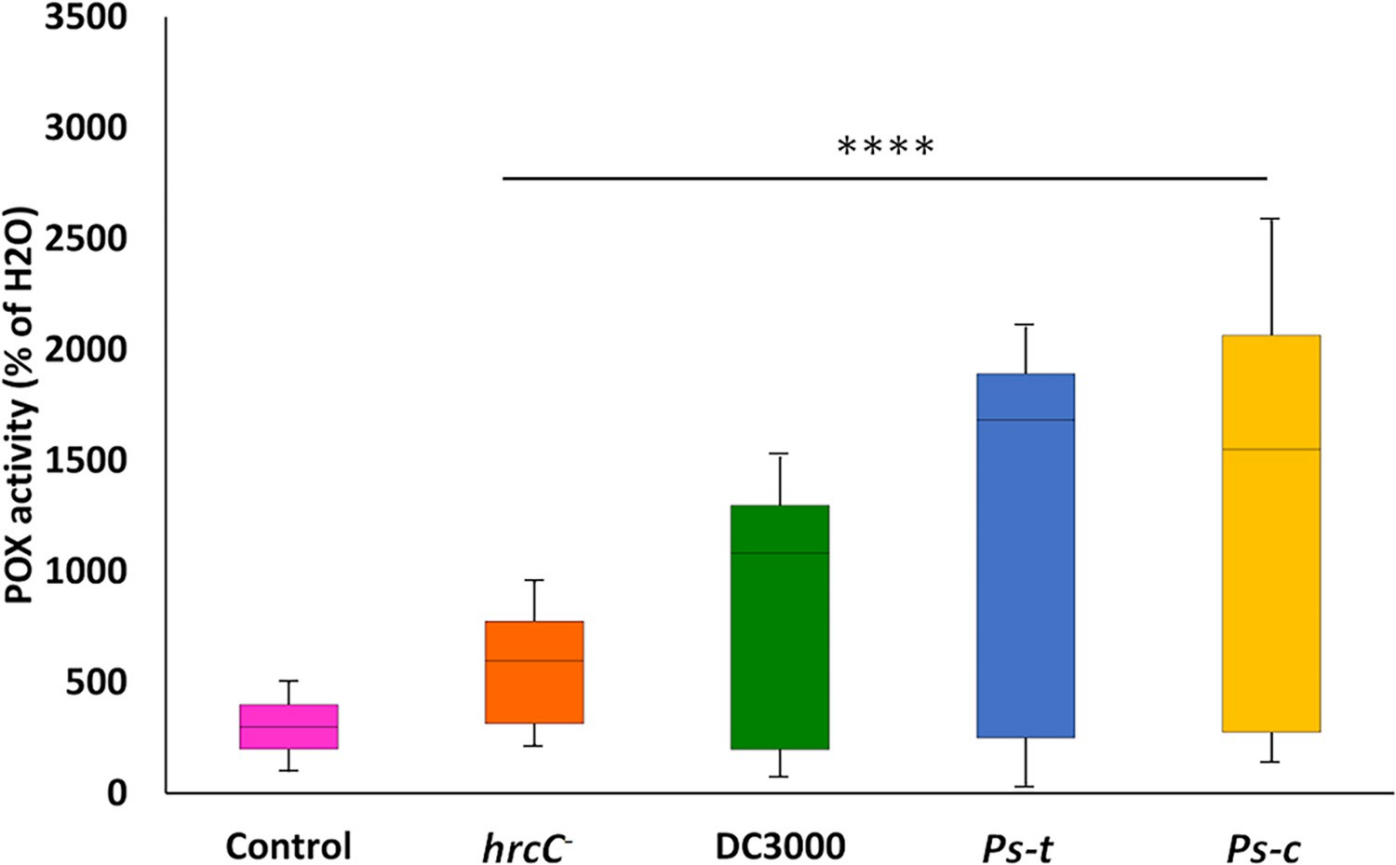
Peroxidase (POX) response of oat seedlings to treatment with *Ps-c*, *Ps-t*, DC3000 and *hrcC* mutant. The assay assesses the activity of POX enzymes generated in response to innate immune activation. Leaf disks from 3-week-old plants (Dunnart cv) were treated with water (control = no bacteria) or with a noted dose (OD_600_ ≈0.3) of the respective *Pseudomonas* bacteria. Total POX activity was measured 20 h after treatment and data is shown as the average of the measured values. Graphs represent data averaged from eleven repeated experiments with 3 biological replicates for each sample (*n* = 33). Error bars represent standard error of the mean, four asterisk (****) indicates *p* < 0.0001, Student t-tests. The lower quartile value, median value, and upper quartile value are shown in boxes, while the whiskers extend to the lowest and highest values.

A 96-well microtiter plate-based assay was used for analysing plant PTI by evaluating the activity of POX enzymes produced in response to bacterial treatment. The experiment was carried out using a fixed concentration of bacteria (OD_600_≈0.3) to assess the kinetics of the plant POX response to the various strains of *Ps*. Unlike the ROS burst as detected by the luminol-based assay, which occurs over minutes, detectable POX activity is not found until 16 h after treatment, with increasing levels seen after 22 h [45]. POX activity increased 9-, 9-, 7-, and 6-fold in the treated (*Ps-c*, *Ps-t*, DC3000 and *hrcC*^*−*^) plants, respectively compared to the control (Fig 3). The highest POX activity was observed for the *Ps-c*-treated plants.

### UHPLC-qTOF-MS

**Fig 4:**
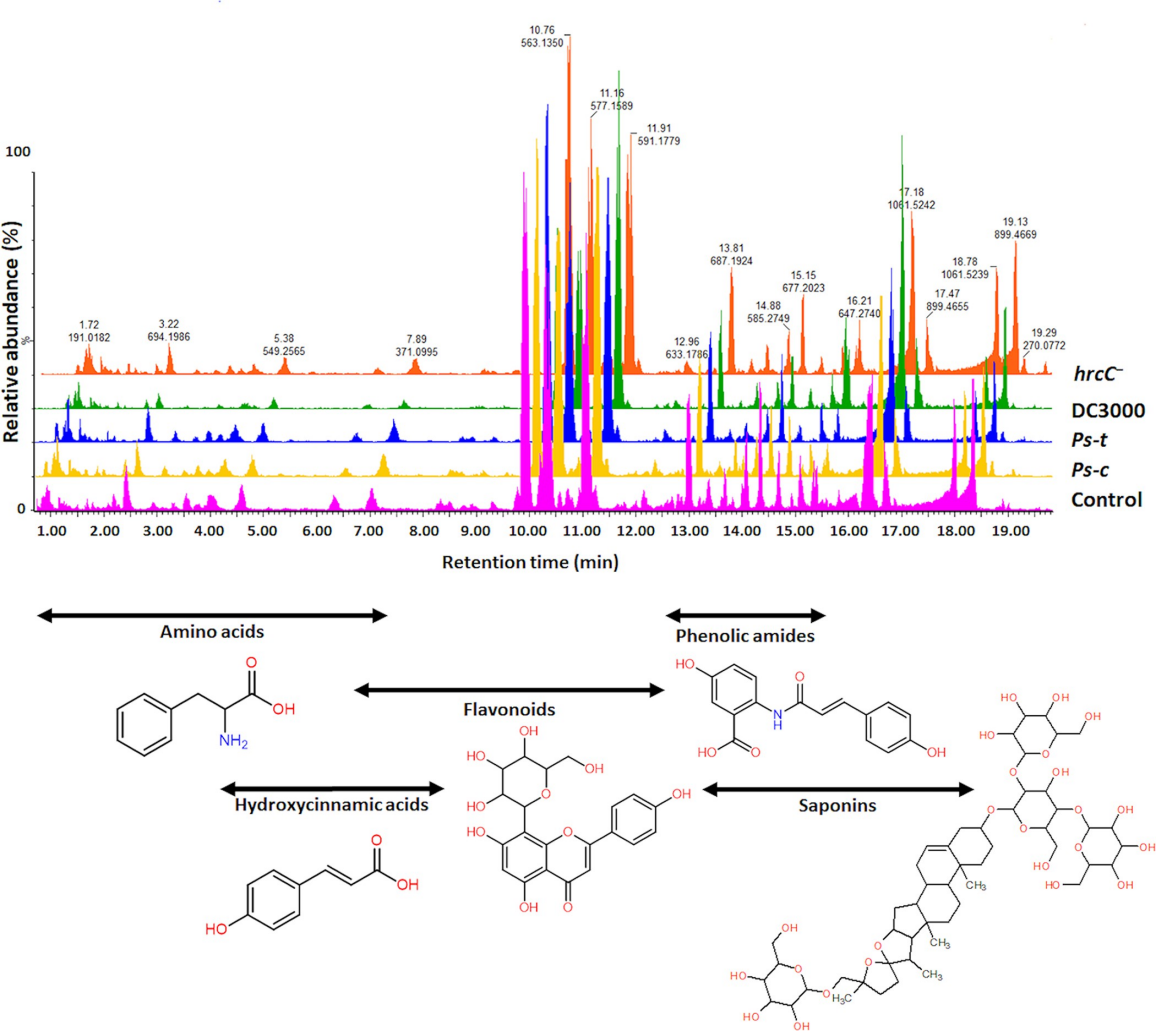
Representative UHPLC-MS base peak intensity (BPI) chromatograms in ESI(−) mode showing the unique metabolite profiles present in leaf extracts of oat seedlings at 2 d.p.i. following treatment with the respective strains of *P*. *syringae* (*Ps-c*, *Ps-t*, DC3000 and *hrcC*^*−*^). The BPI chromatograms illustrate evidently differential peak populations (based on presence and intensities) of *P*. *syringae* infected vs the untreated control. The linked y-axes indicate the relative peak abundance (%) of the metabolite signatures at their respective retention times (min). The chromatograms are staggered along the x-axis. A representative of each metabolite class is presented below the chromatograms, indicating the elution range.

Extracts from leaf tissues were analysed on an UHPLC-qTOF-MS system equipped with an ESI source for the identification of individual metabolites originating from metabolite classes of varied polarity. The data was collected in both ESI (+/–) modes, which was important for the investigation of particular metabolite classes with distinct chemical characteristics. After pathogen treatment, visual inspection of the base peak intensity (BPI) MS chromatograms (Fig 4) in negative ionisation mode revealed distinct variations in peak intensities as well as the presence/absence of peaks across all samples. The chromatograms demonstrate that bacterial infiltration with the different pathovars causes distinct metabolic changes. Initial optimisation tests revealed that the majority of extractable metabolites ionised better in the ESI (–) mode; thus, only these data sets are provided and illustrated further. This section describes an untargeted approach that was used to elucidate and identify as many statistically significant metabolites involved in oat plant defence as possible.

### Chemometric analyses for the elucidation of metabolic changes in response to the different *Pseudomonas syringae* pathovars

**Fig 5:**
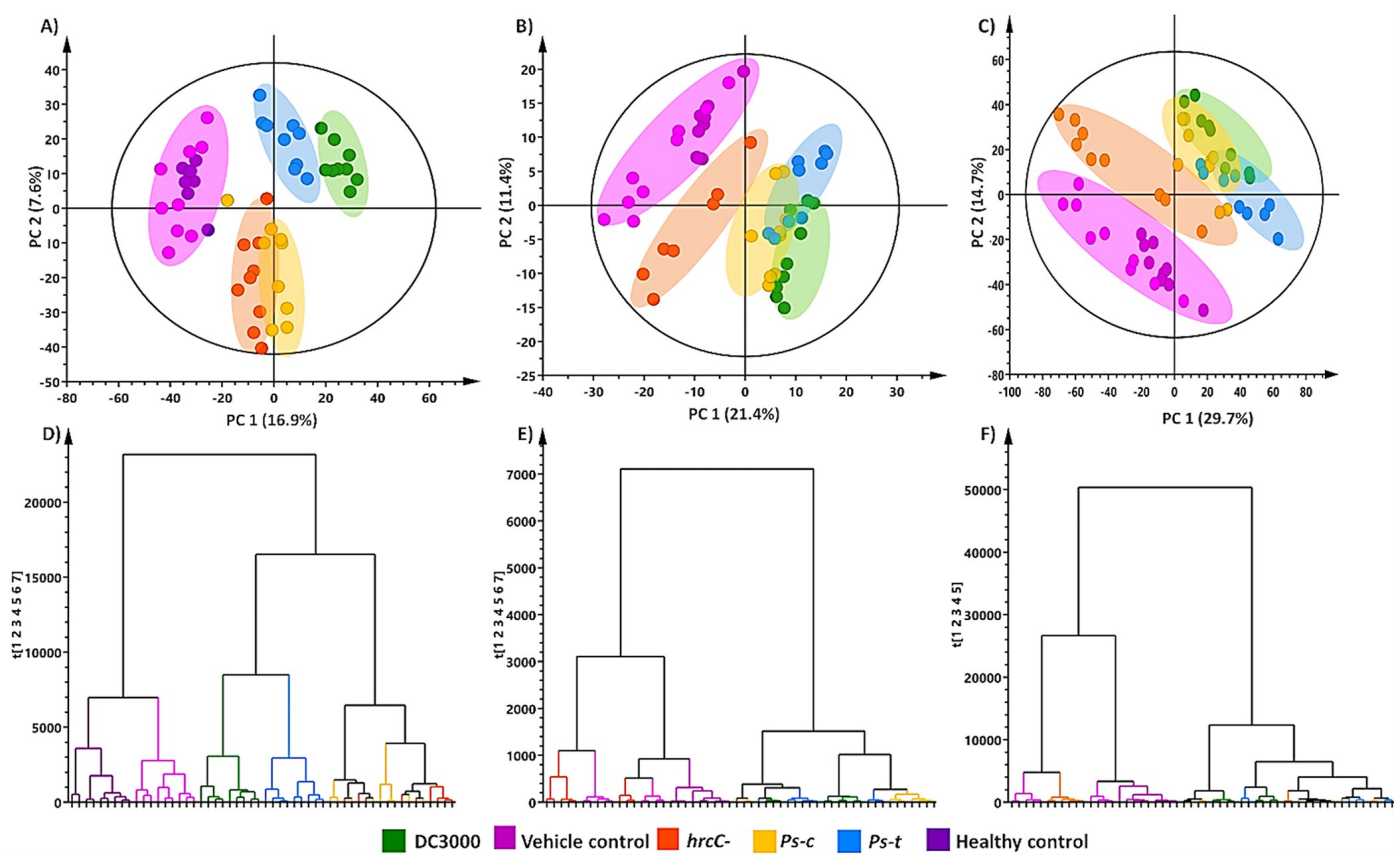
PCA score plots of the UHPLC-MS ESI(−) data of leaf extracts from oat seedlings treated with the respective *P*. *syringae* pathovars and the corresponding HCA dendrograms. Two dimensional PCA scores plots showing differential groupings of the treatments (*Ps-c*, *Ps-t*, DC3000 and *hrcC*^*−*^) and controls at **(A)** 2 d.p.i. **(B)** 4 d.p.i. and **(C)** 6 d.p.i.. VC (vehicle control, light purple) represents plants sprayed with a solution free of bacteria and HC (healthy control, dark purple) the untreated group. The Hotelling’s T2 is illustrated by an ellipse on the score plot indicating a 95% confidence interval. Ward-linkage HCA dendrograms that correlate to PCA plots A, B and C respectively and demonstrates the hierarchical breakdown of the data both before (control) and after treatment at **(D)** 2 d.p.i. **(E)** 4 d.p.i. **(F)** 6 d.p.i.

The unique chromatographic profiles offered a visual evaluation confirming the occurrence of metabolic reprogramming resulting from pathogen treatment. This therefore prompted obtaining more information regarding these underlying changes by data mining and comparative chemometric analyses to reveal more significant structures within the datasets that distinguish the control from the treated conditions. The unsupervised PCA reduces the multi-dimensionality of the dataset and subsequently enables biological interpretability by projecting the data in a lower-dimensional plane, thereby exposing underlying structures, groupings and trends in the data sets. The principal components (PCs) of each model illustrates distinct treatment-related groupings when compared to the control for the Dunnart cultivar at 2-, 4- and 6 d.p.i. (Fig 5A–5C) respectively. Based on the respective PCA plots, HCA plots were constructed as dendrograms that outline the similarities and differences between the individual groups/clusters in a hierarchical format. The dendrograms at the respective time points illustrate that the data clusters into two main branches separating the control and treated groups (Fig 5D–5F). Further separation is apparent with subsequent branches forming within the treated groups at 2 d.p.i. (Fig 5D) showing clusters of DC3000 and *Ps-t vs Ps-c* and *hrcC*^*−*^ treated groups. At 4 d.p.i. overlapping clustering is observed (Fig 5E) for the control and *hrcC*^*−*^ treated groups suggesting similar metabolite content *vs* the other branch showing clustering of DC3000, *Ps-t* and *Ps-c*. A similar trend and grouping is observed at 6 d.p.i. (Fig 5F).

**Fig 6:**
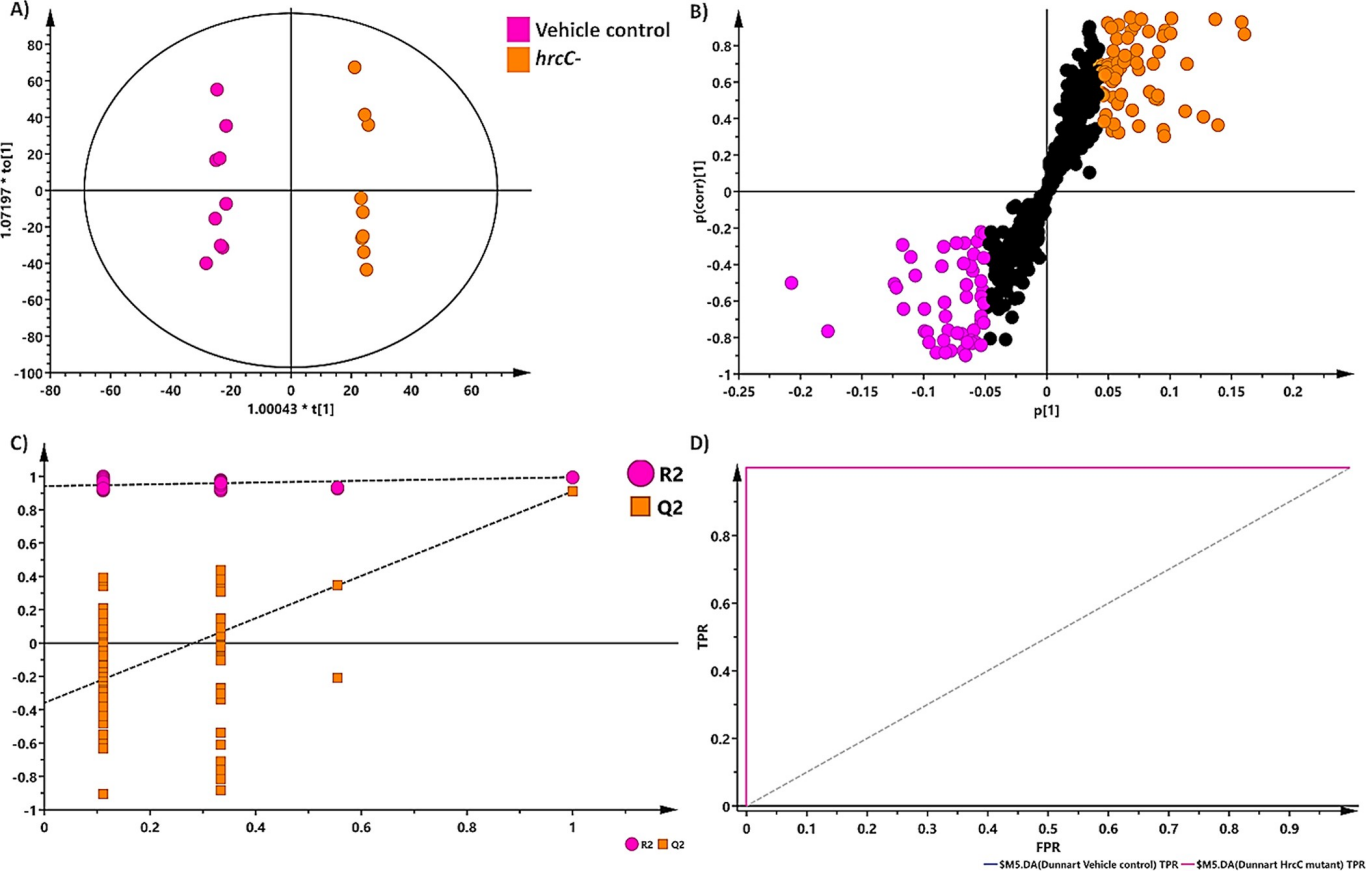
An orthogonal projection to latent structures discriminant analysis (OPLS-DA) model of the control and *hrcC* mutant infected plants. **(A)** An OPLS-DA scores plot summarising the relationship among different datasets to visualise group clustering between the control and infected groups at 4 d.p.i. based on their leaf-extracted metabolic profiles obtained in ESI(–) MS mode (R^2^ = 0.999, Q^2^ = 0.995, CV-ANOVA *p*-value = 1.89349 x 10−^15^). **(B)** The corresponding OPLS-DA loadings S-plot of (A). The pink and orange circles indicate the values situated far out (p[1] > 0.05, < -0.05 and p(corr) >0.3, < -0.3) in the S-plot, representing statistically significant ions that are possible discriminatory variables between the control and infected groups. **(C)** The response permutation test plot (n = 100) for the OPLS-DA model. **(D)** A receiver operating characteristic (ROC) curve summarises the ability of a binary classifier (OPLS-DA), with a classifier having perfect discrimination producing a ROC curve that passes through the top left corner to indicate 100% sensitivity and specificity.

The control and treated groups were then further compared using a supervised discriminant analysis method with an example illustrated in Fig 6A where the OPLS-DA score plot demonstrates differential sample clustering between the control and *hrcC*^*−*^ treated group. To choose discriminating ions between treatments the corresponding loadings S-plot was constructed with an example of the control *vs hrcC*^*−*^ treatment shown in Fig 6B. The S-plot provides a visual explanation of the OPLS-DA model by displaying the properties (*m/z* ions) that contribute to class differentiation. The loadings S-plot assisted in identifying metabolite signatures that appeared statistically significant for the respective treatments. The covariance (magnitude) and correlation (reliability) of the samples in the model are respectively represented on the axes as p[1] and p(corr)[1]. MS spectral-based metabolite identification was used to identify discriminant ions with a |p(corr)| of ≥0.3, ≤0.3 and a co-variance value of |(p1)| ≥0.05, ≤0.05. The reliability of each model was assessed using cross-validation analysis of variance testing (CV-ANOVA) as a diagnostic tool, with models of significance having *p*-values of <0.05 [54]. The permutation test (*n* = 100) revealed that the OPLS-DA models had higher R^2^ and Q^2^ values than the 100 permuted models, implying that the produced models were statistically superior to the permuted models (Fig 6C). The respective computed OPLS-DA models were used as binary classifiers and showed perfect discrimination, with the ROC curve passing through the top left corner indicating 100% sensitivity and specificity (Fig 6D). OPLS-DA models with corresponding loadings S-plots were generated for all treatments along with ROC curves and permutation plots to test the validity of each model. Due to the large number of generated models only one example is shown, however the R^2^X(cum), R^2^Y, Q^2^(cum), and CV-ANOVA determined *p*-values for each generated model are captured in S1 Table.

A list of annotated (putatively identified) metabolites were generated after significant ions were selected from the respective loadings S-plots and presented in Table 1. The experimental section ‘Metabolite annotation and semi-quantitative comparison’ describes how the statistically significant variables were annotated. The potential chemical structures were investigated by examining the fragmentation patterns that were produced under various collision energies MS^E^ (S2 Fig). A total of 51 metabolites were identified for the different treatments and time points. In the table, metabolites are shown that presented as discriminatory for a particular treatment (with a variable importance in projection score, VIP > 1.0) as well as metabolites that were readily identified and had a fold change ≥ 1.5 (indicated with *). The annotated metabolites were also categorised based on the respective metabolite classes which included: fatty acids, amino acids, phenolic acids, phenolic acid amides, steroidal saponins, flavonoids, and alkaloids.

**Table 1:** List of annotated, putatively identified secondary metabolites produced in response to host and nonhost interactions of *P*. *syringae* pathovars on oat leaf tissues. Metabolites were identified according to MSI level 2 guidelines where the accurate mass, fragmentation data, database entries and published literature were used for annotation. These discriminatory metabolites were derived from OPLS-DA S-plots with rigorous statistical validation. VIP scores for all the reported metabolites were > 1.0. Presence or absence as discriminatory metabolites are shown for each treatment.

#	Rt (min)	*m/z*	Putative identification	Molecular formula	*Ps-coronafaciens*			*Ps-tabaci*			*Ps-tomato* DC3000			*Ps-tomato DC3000*, *hrcC*			Control		
Days post inoculation (d.p.i.)					2	4	6	2	4	6	2	4	6	2	4	6	2	4	6
**Phenolic amides**																			
1	13.72	298.070	Avenanthramide A**	C_16_H_13_NO_5_				○	○	○	○	○	○		○	○	○	○	○
2	14.22	328.077	Avenanthramide B**	C_17_H_15_NO_6_		○	○	○	○	○	○	○	○		○	○	○	○	○
3	15.5	324.085	Avenanthramide O/R	C_18_H_15_NO_5_				○	○	○	○	○	○				○	○	○
4	18.13	308.092	Avenanthramide L	C_18_H_15_NO_4_		○	○		○	○	○	○	○		○	○	○	○	○
5	12.98	314.062	Avenanthramide C**	C_16_H_12_NO_6_		○	○		○	○	○	○	○		○	○	○	○	○
6	22.10	312.172	Avenanthramide E*	C_17_H_15_NO_5_	○		○	○		○	○		○	○		○	○	○	○
**Steroidal saponins**																			
7	16.48	1061.522	Avenacoside A**	C_51_H_82_O_23_											○	○			
8	16.76	945.478	26-Desglucoavenacoside A	C_45_H_72_O_18_		○	○		○	○		○	○			○	○	○	○
9	18.42	1225.323	Avenacoside B	C_57_H_92_O_28_	○	○	○		○	○		○	○	○	○	○		○	○
10	18.07	1061.531	26-Desglucoavenacoside B	C_51_H_82_O_23_		○	○		○	○		○	○	○	○	○	○	○	○
**Phenolic acids**																			
11	13.05	193.050	Ferulic acid*(**)	C_10_H_10_O_4_	○	○		○	○		○	○	○	○	○	○	○	○	○
12	6.94	179.034	Caffeic acid*(**)	C_9_H_8_O_4_	○	○		○	○		○	○		○	○	○	○	○	
13	16.47	225.071	Sinapic acid*(**)	C_11_H_12_O_5_	○	○		○	○	○	○	○		○	○	○	○	○	○
14	2.42	315.074	Gentisic acid glucoside*	C_13_H_16_O_9_			○	○	○	○	○		○	○	○	○	○	○	○
15	3.09	337.093	Coumaroylquinic acid*	C_16_H_18_O_8_	○	○	○	○	○	○	○	○	○	○	○	○		○	
16	13.39	369.121	Sinapaldehyde glucoside*	C_17_H_22_O_9_	○	○	○	○	○	○	○	○	○	○	○	○			
17	16.02	371.138	Syringin*	C_17_H_24_O_9_	○	○	○	○	○	○	○	○	○	○	○	○			
18	4.52	385.116	Sinapic acid glucose*	C_17_H_22_O_10_	○	○	○	○	○	○	○	○	○	○			○	○	○
19	18.55	271.082	Naringenin*(**)	C_15_H_11_O_5_													○	○	○
20	1.69	315.069	Protocatechuic acid hexose	C_13_H_16_O_9_			○	○	○	○	○		○	○	○	○	○	○	○
21	17.88	240.067	Benzoylanthranilic acid	C_14_H_11_NO_3_	○	○	○		○	○	○	○	○		○	○	○	○	○
22	7.05	371.098	Dihydroferulic acid 4-O-glucuronide	C_16_H_20_O_10_				○		○	○	○	○	○			○	○	○
23	3.88	367.102	3-O-Feruloylquinic acid*	C_17_H_20_O_9_	○	○	○	○	○	○	○	○	○	○	○	○		○	
**Flavonoids**																			
24	14.71	423.223	Sophoraflavanone G	C_25_H_28_O_6_	○			○	○	○	○	○	○	○			○		
25	11.99	449.149	Auriculoside	C_22_H_26_O_10_		○	○	○	○	○	○	○	○		○	○	○	○	○
26	10.36	577.155	Vitexin 2’’-O-rhamnoside	C_27_H_30_O_14_				○						○	○	○			○
27	12.22	687.193	Tricin ether glucopyranoside	C_33_H_36_O_16_	○	○	○		○	○	○	○	○	○	○	○	○	○	○
28	16.73	435.221	Kanzonol I	C_27_H_32_O_5_	○	○	○	○	○	○	○	○	○		○	○	○	○	○
29	10.6	431.098	Vitexin	C_21_H_20_O_10_	○	○	○	○	○	○	○	○	○						
30	9.82	593.151	Isovitexin 2’’-O-glucoside	C_27_H_30_O_15_	○	○	○	○	○	○	○	○	○						
31	9.93	563.141	Isovitexin 2’’-O-arabinoside	C_26_H_28_O_14_	○			○							○	○			
32	8.36	593.150	Isovitexin -7-O-glucoside	C_27_H_30_O_15_	○	○	○	○	○	○	○	○	○	○	○	○		○	○
33	16.7	413.143	Quercetin dimethyl ether methylbutyrate*	C_22_H_22_O_8_				○		○	○		○				○	○	○
34	11.91	443.154	Isovolubilin	C_23_H_24_O_9_	○	○	○	○	○	○	○	○	○	○			○	○	○
35	21.51	505.254	Isoamoritin	C_31_H_38_O_6_	○	○	○	○	○	○	○	○	○	○	○	○	○		
36	14.04	515.251	Formononetin glucoside malonate	C_25_H_23_O_12_		○	○	○	○	○	○	○	○	○			○	○	
37	11.04	591.172	Acacetin 7-O-rutinoside	C_28_H_32_O_14_				○			○				○	○			○
38	10.38	593.081	Kaempferol-7-O-neohesperidoside	C_27_H_30_O_15_	○			○							○	○			○
39	9.32	633.182	Linarin monoacetate*	C_30_H_34_O_15_		○	○		○	○		○	○	○	○		○	○	○
40	15.7	677.206	Prenylkaempferol diglucoside	C_32_H_38_O_16_	○	○	○		○	○	○	○	○	○	○				
41	11.82	429.175	Ononin	C_22_H_22_O_9_	○	○	○	○	○	○		○	○	○	○	○	○	○	○
**Fatty acids**																			
42	16.55	327.217	Linoleic acid	C_18_H_32_O_5_	○			○				○	○	○	○	○	○	○	○
43	22.06	559.312	Di-rhamnosyl linolenic acid	C_28_H_48_O_11_	○			○	○	○	○	○	○	○					
44	20.95	325.201	9-Oxo-12,13-dihydroxy-10E,15Z-octadecadienoic acid*	C_18_H_30_O_5_		○	○			○	○		○		○	○	○	○	
45	21.64	293.211	Hydroxylinolenic acid*	C_18_H_30_O_3_		○	○		○	○		○	○				○		
46	21.85	723.379	Palmitoleic-linoleic-glucoside*	C_33_H_36_O_16_	○	○	○			○	○	○	○	○	○	○	○	○	○
47	12.01	415.196	Ethyl 7-epi-12-hydroxy-jasmonate glucoside	C_20_H_31_O_9_		○	○	○	○	○				○	○	○	○	○	○
**Amino acids**																			
48	2.44	203.081	Tryptophan**	C_11_H_12_N_2_O_2_	○			○						○	○			○	○
**Alkaloids**																			
49	1.68	166.043	Hordenine*(**)	C_10_H_15_NO		○	○	○	○		○	○	○	○	○	○	○	○	○
50	6.34	351.128	Feruloylserotonin*	C_20_H_20_N_2_O_4_	○	○	○	○		○	○	○	○	○	○	○		○	○

(*) Metabolites that were detected and identified in the respective treatments but did not present as discriminatory—shown are the presence at the respective time points where fold change was calculated to be ≥ 1.5.

(**) Metabolite identity was confirmed with an authentic analytical standard–MSI level 1.

(○) Indicates metabolites that did not appear as distinguishing ions in the corresponding treatments or time points.

### Time-related profiling of oat plants upon treatment with pathovars of *Pseudomonas syringae*

**Fig 7:**
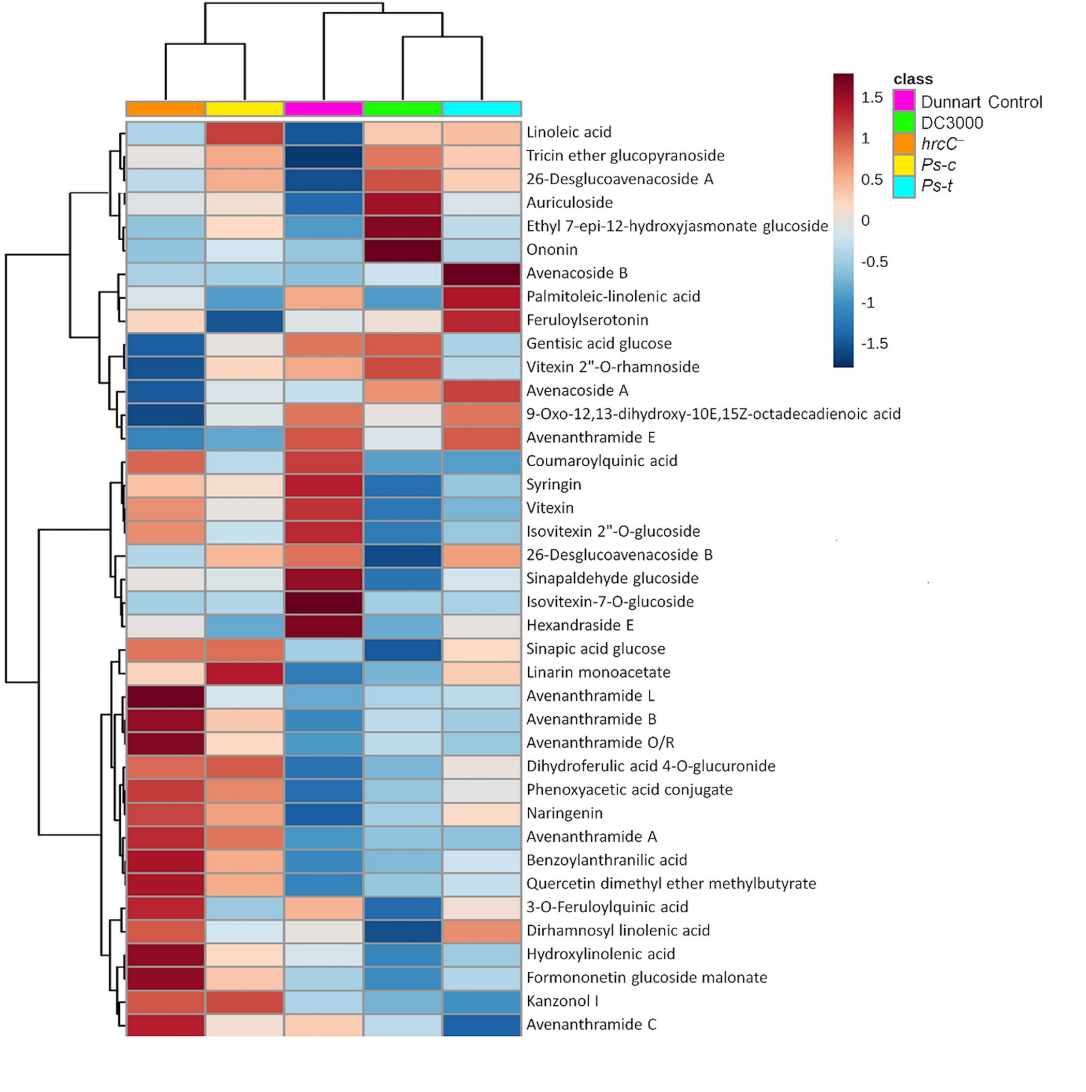
Heatmap trends of the relative concentrations of the annotated discriminatory metabolites (Table 1). The heatmap illustrates the relative intensities using colour intensity to depict the ions detected in each sample. The respective infected and control groups are indicated on the map, which was created using the Pearson distance and Ward’s linkage rule. The mean peak intensities of each detected metabolite are shown following Pareto scaling of the data. Brown is used to indicate values that are higher than average, and blue is used to indicate values that are lower than average. Each row represents discriminant metabolites, and the columns represent treatment groups.

In order to gain more insight into the metabolic reprogramming that occurs in the leaf tissue of the oat seedlings in response to treatment with the *P*. *syringae* pathovars, biochemical interpretations were drawn from the putatively annotated metabolites (Table 1). In addition to the amino acids and fatty acids which are classified as primary metabolites, phenolic acids and phenolic amides, saponins, flavonoids, and alkaloids were among the metabolite groups identified as secondary or specialised metabolites. Changes in these metabolite classes were analysed from a metabolome perspective and revealed alterations involved due to the bacterial treatments. The presence and amount of the respective metabolites in the various treatment and control groups were considered using data visualisation tools available on MetaboAnalyst 5.0. A heatmap was constructed using the average integrated peak areas of the different metabolites (Fig 7). The infographic clearly distinguishes between the treated and control groups. The distinguishing characteristics are evidently illustrated among the respective treatments. The profile could be beneficial in identifying metabolic markers linked with disease resistance in oat plants against the respective pathovars of *P*. *syringae*. By profiling these host and nonhost responses deeper insight can be gained into oat plant defence mechanisms.

**Fig 8:**
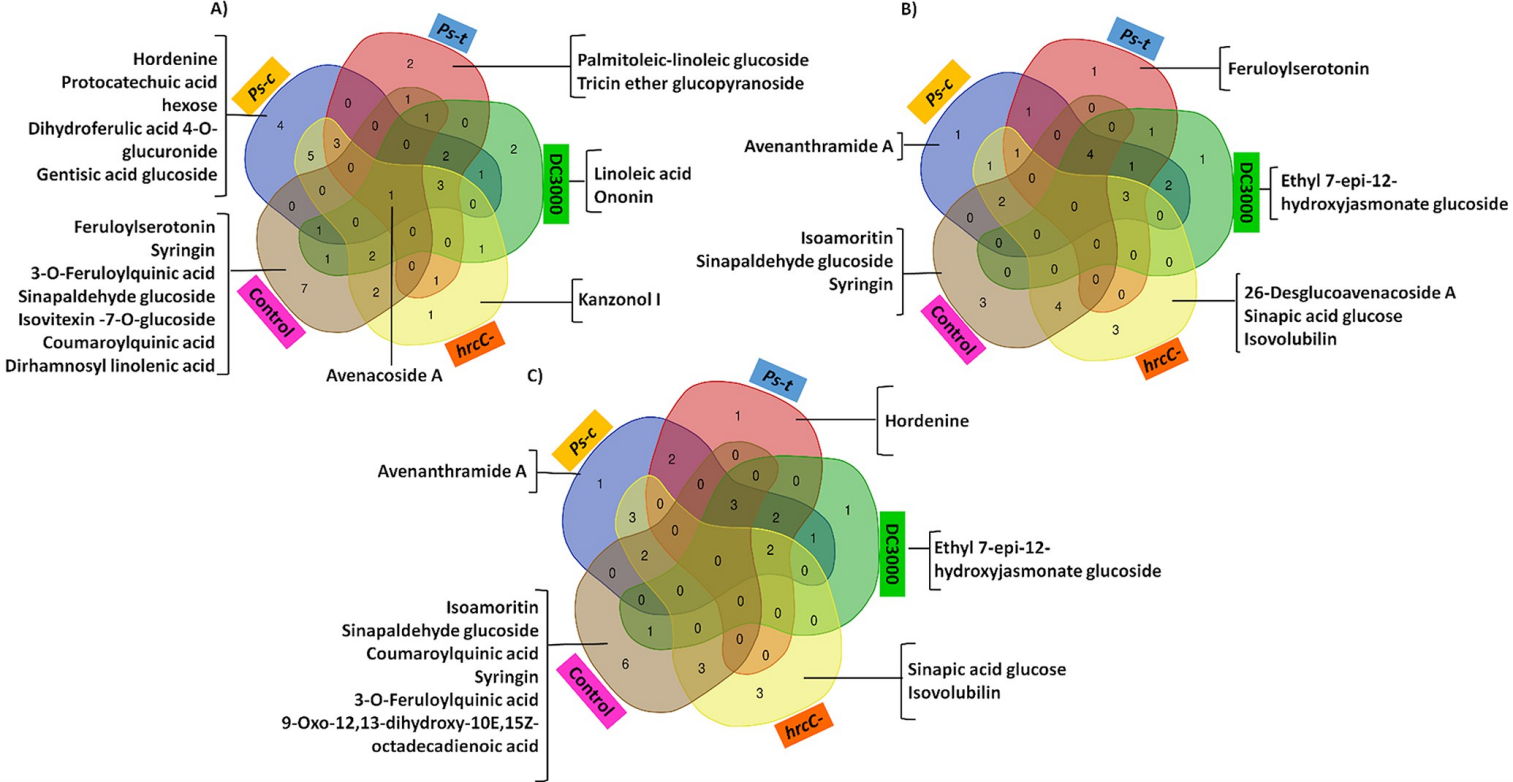
Venn diagrams illustrating the partial overlap and differences of the identified metabolites among the respective treatments of the oat seedlings by the *P*. *syringae* pathovars. The infected and control groups are compared. The numerical values indicate metabolites (Table 1) that are unique to, and also shared among the treatments at the respective time points. **(A)** 2 d.p.i. **(B)** 4 d.p.i. **(C)** 6 d.p.i.

Venn diagrams were constructed to highlight overlapping and unique metabolites. Fig 8 represents Venn diagrams constructed at the respective time points 2- (Fig 8A), 4– (Fig 8B) and 6 d.p.i. (Fig 8C). Unique metabolites presented for the respective groups and time-specific metabolites were noted for certain treatments. Starting with 2 d.p.i., 7 metabolites were found as unique for the control group (feruloylserotonin, syringin, 3-O-feruloylquinic acid, sinapaldehyde glucoside, isovitexin-7-O-glucosde, coumaroylquinic acid and dirhamnosyl linolenic acid). At 4- and 6 d.p.i. only 3 (namely isoamoritin, a triprenylated flavanone, sinapaldehyde glucoside and syringin) and 6 (isoamoritin, sinapaldehyde glucoside, coumaroylquinic acid, syringin, 3-O-feruloylquinic acid and 9-oxo-12,13-dihydroxy-10,15-octadecadienoic acid) metabolites presented as unique to the control at these time points respectively. The only common metabolites that presented across the three time points were syringin and sinapaldehyde glucoside. The *Ps-c*-treated group revealed hordenine, protocatechuic acid hexose, dihydroferulic acid 4-O-glucuronide and gentisic acid glucoside as signatory metabolites at 2 d.p.i. At 4- and 6 d.p.i. only avenanthramide (Avn) A presented as unique for this treatment. Interestingly, for the *Ps-t* treatment each time point presented different metabolites, at 2 d.p.i. (palmitoleic-linoleic glucoside and tricin ether glucopyranoside), 4 d.p.i. (feruloylserotonin) and 6 d.p.i. (hordenine). The DC3000 treatment showed linoleic acid and ononin (a isoflavone glycoside) as signatory features at 2 d.p.i. and ethyl 7-epi-12-hydroxyjasmonate glucoside at 4- and 6 d.p.i.. Treatment with the *hrcC* mutant revealed the prenylated isoflavonoid kanzonol I as signatory at 2 d.p.i., whereas 3 metabolites presented at 4 d.p.i. (26-desglucoavenacoside A, sinapic acid glucose and isovolubilin, an isoflavone rhamnoside) and 6 d.p.i. (sinapic acid glucose and isovolubilin). The only shared metabolites for this treatment being sinapic acid glucose and isovolubilin at 4- and 6 d.p.i.. Among the control and all the treated groups, only one common overlapping metabolite was noted at 2 d.p.i. (avenacoside A).

Among the four treated groups, 3 metabolites overlapped for 2- and 4 d.p.i. namely 26-desglucoavenacoside A, naringenin, hydroxylinolenic acid for 2 d.p.i. and Avn E, quercetin dimethyl ether methylbutyrate and naringenin for 4 d.p.i.. At 6 d.p.i., naringenin and tryptophan were present as unique for the four treated groups with naringenin (as the precursor of flavonoid metabolites) being the common overlapping metabolite for all the treatments across all time points. Other inferences that can be drawn from the Venn diagrams without mentioning all the specific overlapping metabolites, is that among the respective groups the greatest overlapping responses are seen among the *Ps-c* and *hrcC*^*−*^ treated groups. Some of the key metabolites that overlap for these two treatments are Avns A, B and O/R at 2 d.p.i. and Avn O/R being commonly present across all time points.

**Fig 9:**
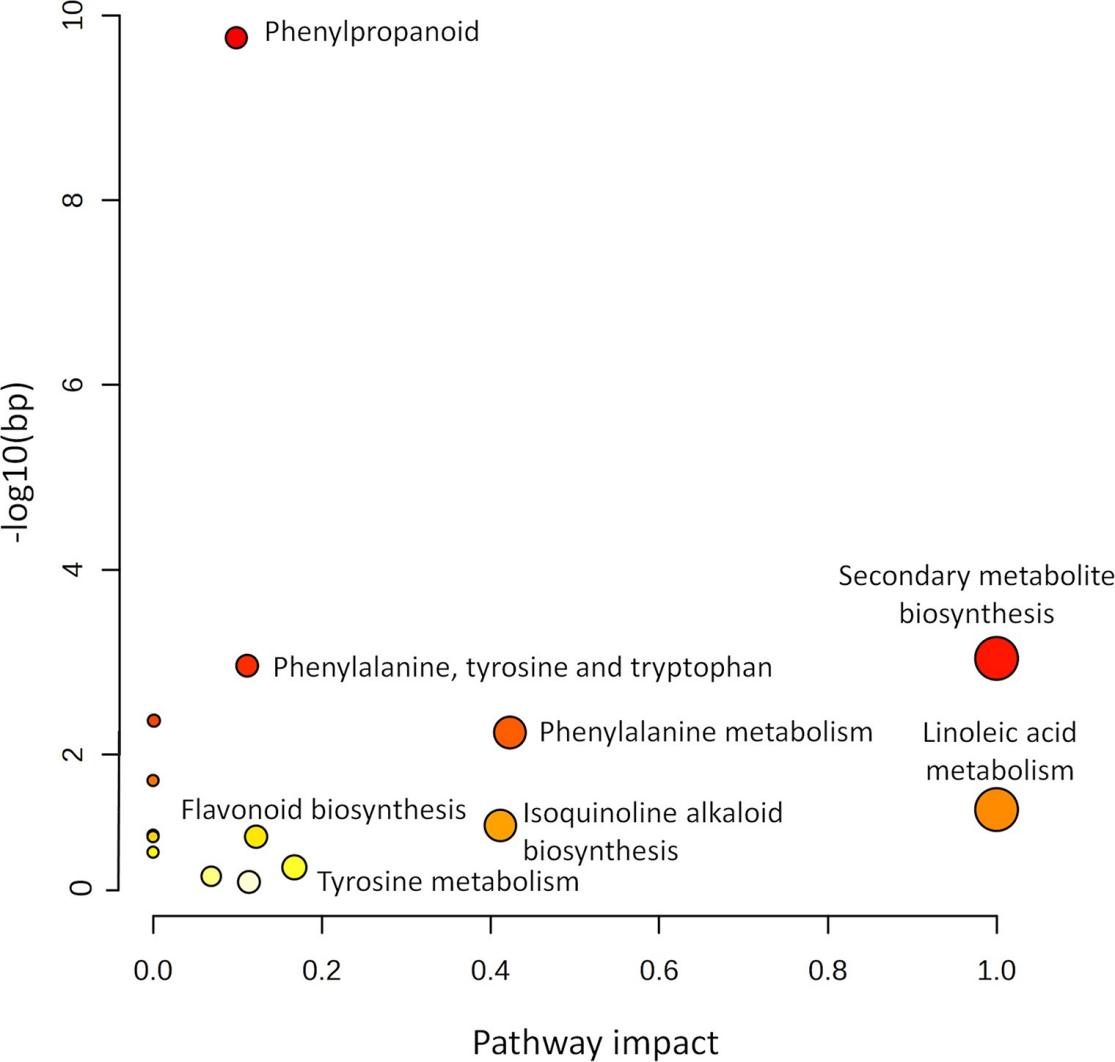
Pathway analysis summary of all MetaboAnalyst-computed metabolic pathways displayed according to their significance or pathway impact. The diagram depicts all the matching pathways, organized by *p*-values (y-axis; pathway enrichment analysis) and pathway impact values (x-axis; pathway topology analysis). The impact values define the node sizes, and each node is coloured according to its matching *p*-value. The graph thus indicates the general secondary metabolic biosynthetic pathways as having the greatest impact with the phenylpropanoid pathway as having the greatest significance.

These defence-related metabolites interact with one another through various metabolic pathways rather than acting independently. Metabolic pathway mapping was thus carried out to elucidate the most pertinent pathways implicated in oat responses to inoculation with the respective pathovars of *P*. *syringae*, which will aid in the biochemical interpretation of the post-infection metabolic perturbations. MetaboAnalyst 5.0 was used to carry out the Metabolomics Pathway Analysis (MetPA). This extremely sensitive web-based application is helpful when it comes to analysing and visualising metabolomic data and can pick up small variations between various compounds. Relative concentration variations of compounds with known KEGG or HMDB identifiers can be used to construct these biological pathways [52,55]. The computed metabolic pathways are presented according to significance or pathway impact as shown in Fig 9. The most significant pathways (displayed on the y-axis) were the phenylpropanoid—and phenylalanine, tyrosine and tryptophan metabolism pathways, whereas the most impactful (displayed on the x-axis) were the linoleic acid pathway and (in general) pathways involved in the biosynthesis of secondary metabolites.

**Fig 10:**
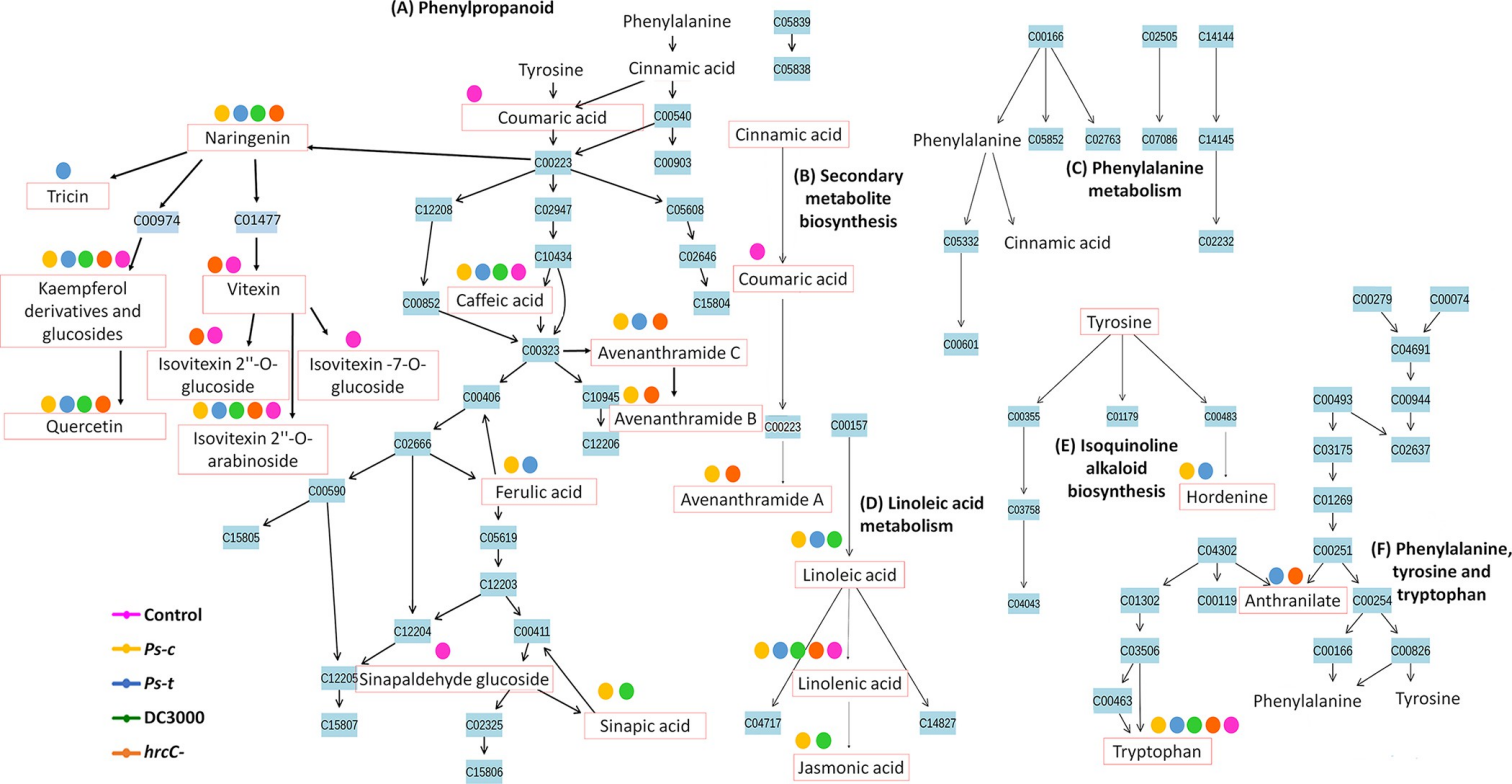
Pathways flagged from metabolome analysis of leaf extracts of oat seedlings treated with pathovars of *P*. *syringae*. Signatory metabolites involved in each pathway are illustrated in red boxes. (**A**) The phenylpropanoid pathway, (**B**) the secondary metabolite pathway overlapping with the phenylpropanoid pathway, and (**C**) phenylalanine metabolism. (**D**) The linoleic acid pathway that showed a high impact after pathway enrichment analysis, along with the secondary metabolite biosynthesis pathway. (**E**) The isoquinoline alkaloid biosynthesis pathway. (**F**) Phenylalanine, tyrosine and tryptophan metabolism. All annotated metabolites (Table 1) could not be mapped due to limitations in the MetaboAnalyst software. Some extensions have been manually added to the pathways (using KEGG as a guideline) to represent more of the annotated metabolites and the pathways they are involved in. Each of the coloured circles corresponds to the respective treatments (*Ps-c*, *Ps-t*, DC3000 and *hrcC* mutant) or control, illustrating where these metabolites presented as signatory.

Some of the most frequently occurring secondary metabolites that are involved in plant growth and defence against abiotic and biotic stressors includes phenolics, flavonoids, coumarins, and lignins that are produced *via* the phenylpropanoid pathway [56]. The phenylpropanoid—and general secondary metabolite biosynthesis pathways show overlap where the conversion of phenylalanine to *p*-coumaroyl-CoA can be seen. Coumaroyl-CoA (KEGG ID = C00223) is an important intermediate in the synthesis of a multitude of secondary metabolites such as the respective flavonoids and the phenolic amides. The presence and distribution of these substances, from both pathways, at the cellular, tissue, and organ levels across the plant kingdom emphasises the myriads of biological and biochemical processes that are vital to the survival of plants [57]. It is well known that these phenolic acids and derivatives such as phenolic amide conjugates can act as naturally occurring antibiotic molecules (pre-existing phytoanticipins or inducible phytoalexins) in plant-pathogen interactions [58,59]. Unsaturated fatty acids (linoleic acids, C18:2) are abundant in plant membranes, which makes them crucial for plant cell structure and function and supportive of adaptive responses through membrane remodelling [56]. In addition, they play a role in the formation oxylipins and signalling molecules such as 12-oxo-phytodienoic acid (OPDA). OPDA in turn acts as precursor of jasmonic acid (JA) and derivatives, which are produced in response to tissue damage brought on by insects, pathogens, herbivores, or mechanical stress [60–62] (Fig 10).

## Discussion

Surface and intracellular recognition are all components of the host’s immune surveillance with significant convergence and reciprocal interplay between PTI and ETI. Effector-triggered susceptibility (ETS) is the outcome of pathogens being able to inject suppressors into plant cells to overcome activated plant defences [63]. In this molecular arms race, both the pathogen and the host go through natural selection to diversify their effectors and R proteins [64]. ETI is the cornerstone of traditional gene-for-gene resistance and is initiated by the recognition of effector or Avr proteins by corresponding plant R proteins. Upon recognition the majority of R proteins participate in the initiation of the HR by producing ROS [65]. As such, the boundary between ‘susceptibility’ or ‘resistance’ (i.e., non-effective or effective nonhost resistance) is frequently an equilibrium between these two states that might be perturbed by the gain or loss of individual genes in the host or the microbe [25,66]. In general, the phenotypic outcome would be classified as resistant, tolerant, or susceptible based on the sum of PTI and ETI, minus inhibitory effects from ETS (also considering the timing and intensity of responses). The development and severity of symptoms in oat crops vary depending on the plant-pathogen interaction and environmental factors [67]. Since the oat plants were infected in a controlled environment, the symptomatic differences reflect responses to the infection with the respective pathovars. It is therefore critical to determine the precise variety or cultivar, as significant differences can occur within cultivars of the same species in terms of susceptibility or resistance/tolerance to various diseases [68]. To resist changing environmental and pathogenic threats, plants rely on an inherent sophisticated and multi-layered innate immune system [69]. As a result, identifying the metabolic phenotypes associated with oat defence responses to infection with the respective *Ps* pathovars would reveal more information about the cellular and metabolic pathways involved in the plant-pathogen relationship [70].

A variety of studies have reported important factors involved in plant nonhost responses such as ROS [71], one of the earliest observable defence strategies in plants. One of the first studies on the role of ROS was done on type II nonhost responses [72] and showed an accumulation of H_2_O_2_ as a strong form of resistance against bacteria. ROS are known to have two essential roles in plant defence to infection, one being the accumulation leading to a HR at the infected site and inhibiting further pathogen growth. The second role is the activation of resistance genes [73,74]. In a study by Smith and Heese [75] the early production of ROS in Arabidopsis plants treated with the DC3000 and *hrcC* mutant strains of *P*. *syringae* pv. *tomato* was evaluated. The ROS production showed similar responses in terms of timing, intensity and duration. Similarly, the timing, intensity and duration of ROS production for both these treatments in this study also showed no significant differences. The triggered ROS production clearly functions independent of the T3SS, as evidenced by the lack of statistically significant differences between the two strains. The ROS response observed in this investigation is thus caused by PTI-dependent processes, which are consistent with effectors being transported into host cells at a much later time post infection. Peroxidases are another important component of plant defence and are activated in host plant tissues by pathogen infection to prevent cellular spread of infection by creating structural barriers (e.g., lignification using mono-lignols as substrates) or by releasing large amounts of ROS to create toxic conditions [76]. The POX assay used measures activity of POX enzymes produced in response to activation of plant innate immunity and is purportedly a marker of PTI [45]. The results in this study showed increases in POX activity in the treated plants as compared to controls. Such increases in POX activity signify increases in levels of lignin formation, suberisation and the HR [77]. Due to POXs being able to operate as both ROS producers and catalytic enzymes depending on a variety of factors such as the availability of substrates and a range of other reaction conditions, it becomes difficult to identify the enzyme responsible for the observed POX activity. However, due to the initial washing phase of the assay, it is unlikely that the POX activity is associated with the ROS burst since any activity would be lost during this step, therefore it is more likely to be involved in cell-wall reinforcement.

The result/outcome of the host response to an attempted infection may be affected by qualitative and quantitative variations, in particular metabolites or classes of metabolites within wider metabolomic profiles. Since the metabolites were seen to accumulate in the leaves in various amounts and with distinct accumulation patterns, it implies that differential reprogramming has occurred over time due to the respective treatments. High or low accumulation at particular time points signify early-, late-, or oscillatory reactions. According to the time-dependent reprogramming, infected plants modify their metabolomes toward inducible defence responses to restrict pathogen entry and further multiplication.

The extensively researched host resistance is frequently referred to as gene-for-gene resistance and is typically extremely specific to a particular genotype or cultivar of plant and against a certain pathogen. Nonhost resistance, on the other hand, is a broad-spectrum resistance displayed by the entire plant species against a given disease and is not pathogen specific. Nonhost resistance is multi-tiered with many barriers reliant on a specific host to prevent pathogen colonisation [7]. These barriers range from preformed barriers and phytoanticipins, to induced defence responses like the HR, lignin accumulation, production of antimicrobials (phytoalexins), and induction of pathogenesis-related (PR) proteins [24,66,78]. Relevant to the evaluation of the results of this study, nonhost resistance has been described ‘as a gradual transitional phenomenon that is influenced by various exogenous factors and that there is ‘a continuum between nonhost and host plants, with several possible intermediate forms’ (e.g., ‘marginal/near/intermediate host’ and ‘near/intermediate nonhost’ or ‘apparent nonhost’) [66].

**Fig 11:**
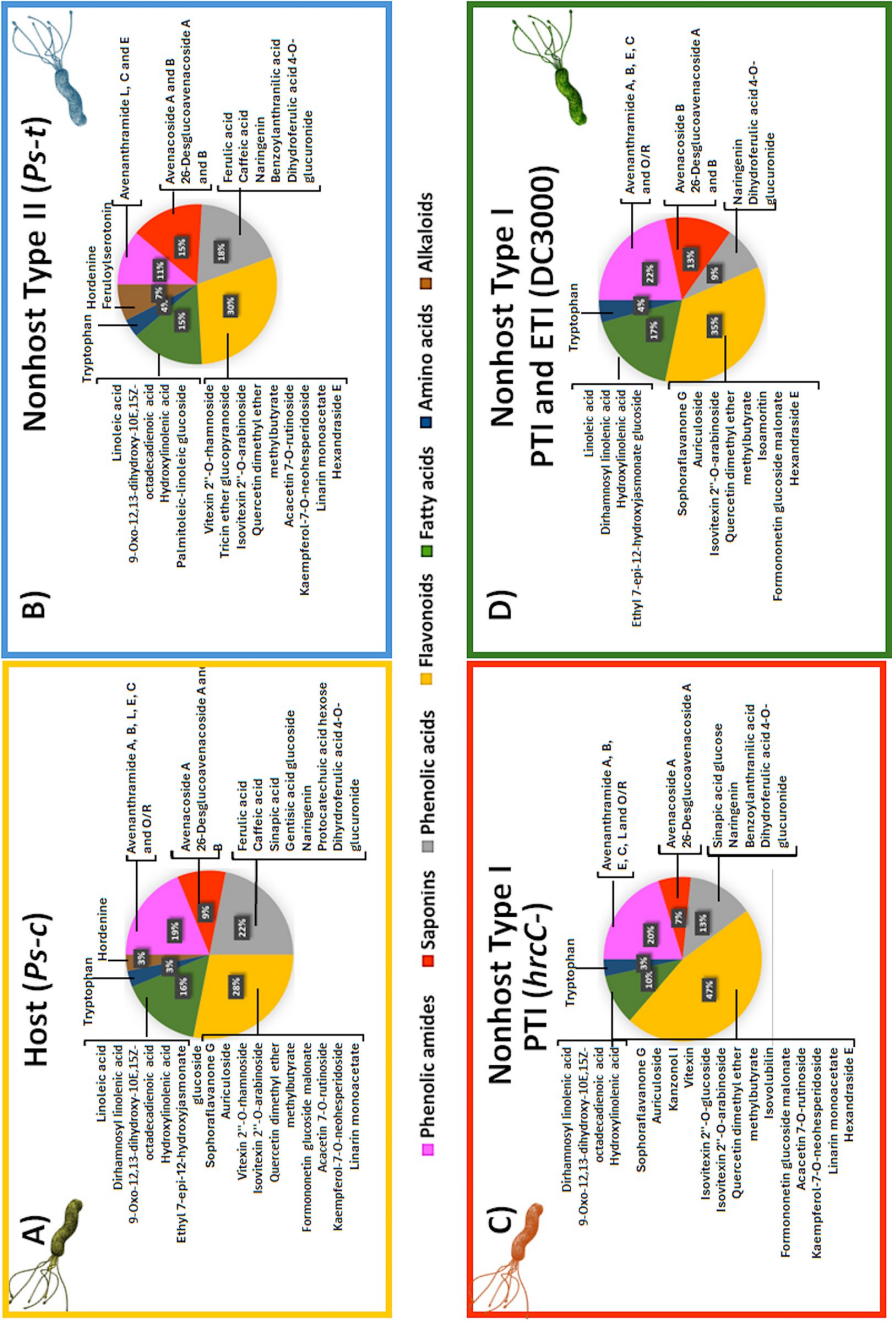
The distribution of the identified metabolite classes across the respective treatments. This figure summarises differences caused by changes in the underlying metabolic profiles of the oat seedlings treated with **(A)** *Ps-c* (host response), **(B)** *Ps-t* (type II nonhost response) **(C)** *hrcC* mutant and **(D)** DC3000 (type I nonhost response) respectively. Each pie chart illustrates where a greater number of discriminatory metabolites were identified for each class.

In this study, an untargeted metabolomics approach has been applied to highlight the metabolic reprogramming that occurs in the oat seedlings (Dunnart cultivar, tolerant to *Ps-c*) under treatment with different pathovars of *P*. *syringae*. Among these, *Ps-c* is known to cause a host response while *Ps-t*, DC3000 and the *hrcC* mutant elicit a nonhost response (*hrcC* encodes a putative outer membrane protein that is conserved in all T3SS). Among the nonhost responses, *Ps-t* can further be described as showing a type II response since symptoms indicative of an HR appeared on leaves treated with this particular pathovar. DC3000 and its *hrcC* mutant on the other hand showed a response that can be categorised as a type I nonhost response. A mRNA profiling study of compatible and incompatible Interactions of Arabidopsis with *P*. *syringae* strains demonstrated that a big portion of the difference between incompatible and compatible interactions can be explained quantitatively with a saturating response curve model, where the plant response in an incompatible interaction was strong but that of a compatible interaction was not [79]. Accordingly, by comparing the responses triggered by DC3000 and the *hrcC* mutant, a metabolic profile can be elucidated that reflects the outcome of effector proteins on the plant defence response (i.e., PTI *vs* PTI and ETI). The metabolites identified (phytoanticipins and phytoalexins) were categorised into classes of phenolic acids and—amides, saponins, flavonoids, and alkaloids. The summary figures below show the distribution of these metabolite classes among the different treatments (Fig 11).

The study revealed phenolic amides as discriminatory metabolites, unique in the treated groups compared to the controls. These metabolites, also sometimes referred to as hydroxycinnamic acid amides (HCAAs), are secondary metabolites that are widely distributed throughout the plant kingdom. The structure of these metabolites is often characterised by the association of at least one hydroxycinnamic acid derivative linked *via* an amide bond to an aromatic mono- or an aliphatic polyamine [80,81]. The most commonly found hydroxycinnamic acids in nature include *p*-coumaric -, caffeic -, ferulic—and sinapic acids along with some glycosylated forms. In oat crops, these phenolic amides consist of an anthranilic acid bound to a hydroxycinnamic acid and named as avenanthramides (Avns) when dimerised [60]. The two precursor metabolites involved in the synthesis of Avns are *p*-coumaric acid that forms *p*-coumaroyl-CoA (early phenylpropanoid pathway) and caffeoyl-CoA that is converted to feruloyl-CoA in the general secondary metabolite biosynthesis pathway (Fig 10). The enzymatic synthesis of *p*-coumaric acid by the stress-responsive phenylalanine ammonia lyase (PAL) from phenylalanine and cinnamate-4-hydroxylase (C4′H) or directly from tyrosine by tyrosine ammonia lyase (TAL), initiates the Avn biosynthesis. The enzyme hydroxyanthranilate N-hydroxycinnamoyl transferase (HHT) catalyses the condensation of 5-hydroxyanthranilic acid with *p*-coumaric acid to form Avn A after (4CL) converts *p*-coumaric acid into its activated CoA thioester. On the other hand, the *p*-coumaroyl-CoA is frequently transformed to *p*-coumaroyl shikimate or quinate before being hydroxylated to generate caffeoyl-CoA by the *p*-coumaroyl-CoA ester 3′-hydroxylase. In the presence of HHT, the caffeoyl-CoA is then condensed with 5-hydroxyanthranilic acid to create Avn C. The caffeoyl-CoA O-methyltransferase (CCoAOMT) enzyme then methylates Avn C to produce Avn B [36,82–84]. To date, over 40 different types have been reported in leaves and grains of oat plants based on their structure [85,86]. The most abundant Avns are A, B and C. These metabolites were identified as discriminatory in the *Ps-c*, *hrcC*^*−*^ and DC3000 treated groups (Fig 11A–11C). Other less abundant Avns were also identified as discriminatory and included Avns O/R, L and E (*Ps-c* and *hrcC*^*−*^). Avns are produced in response to pathogen infection or when oat leaves are treated with various elicitors. In oat, these Avns function as phytoalexins that are induced to act as chemical defence barriers and substrates for cell wall reinforcement upon exposure to pathogens. Additional to the antimicrobial activity, Avns are also potent antioxidant and radical scavenging compounds due to the cinnamic acid structure and the hydroxyanthranilic acid moiety [87,88]. A greater variety of Avns were detected as discriminatory in the host response to *Ps-c* compared to the nonhost response to *Ps-t*. When infected with the *hrcC* mutant and the DC3000 strain (type I nonhost response), the cultivar presented a multitude of Avns. Since these metabolites are produced in all treatments (albeit with differences in apparent quantitative levels), it is suggested that the initial PAMP recognition is enough to trigger the production of these metabolites as the majority of the identified Avns were present as discriminatory ions for the *hrcC* mutant treated group.

Avenacoside A and B and their biologically active counterparts (26-desglucoavenacoside A and B) were detected as discriminatory between the treated and control groups. Avenacosides are saponin phytoanticipins that are physiologically inactive until they are converted by avenacosidase into biologically active 26-desglucoavenacosides in response to tissue damage or pathogen infection [89]. Here, avenacoside A and B can be seen as showing a decrease in level from the control to the infected groups, with 26-desglucoavenacoside A and B increasing in the treated groups. Since the conversion to the physiologically active form was noted in all the treatments, it is evidence that the respective pathovars elicited a clear defence response. These compounds are important in plant defence and have been greatly studied due to their antifungal properties where they are able to bind with sterols in the pathogen membrane and disrupt its integrity, which is the primary mechanism of action against the pathogen. As a result of saponin aggregation with sterol groups, this mechanism is thought to lead to the formation of transmembrane pores, which ultimately leads to cell death [89–91]. It was proposed that similar saponins may also interfere with the bacterial outer membrane’s permeability [91]. Lipopolysaccharides covers over 90% of the surface of Gram-negative bacteria’s cell walls. The study therefore proposed that the lipid A component of lipopolysaccharides may interact with the saponin thereby increasing the permeability of the bacterial cell wall as a result.

Phenolic acids are produced and accumulate in plant tissues in response to stress and/or pathogen attack, where they act as protective agents against invading pathogens. These phenolics are often present as simple glycosides, conjugates to esters or amides or linked to the cell wall; and exhibit insecticidal, antimicrobial, antioxidant, and free radical scavenger activities [59,92]. Depending on their chemical composition, they can be classified as simple phenolics, tannins, coumarins, flavonoids, chromones, xanthones, stilbenes, or lignans. These chemicals are mostly synthesised from shikimic acid as a precursor compound by the phenylpropanoid- and/or malonate pathway. The shikimic acid pathway is used to produce phenylalanine, leading to (hydroxy)cinnamic acids, and their derivatives, including coumarins, lignans, and simple phenols through deamination, hydroxylation, and methylation [93,94]. Here a greater number of phenolic acids were present in the *Ps-c* and *Ps-t*-treated groups. More flavonoids were however present in the *hrcC*^*−*^ and DC3000-treated groups. The role of flavonoids in the tomato-DC3000 interaction was evaluated and revealed that these compounds may operate as the plant’s first line of defence against pathogen invasion by preventing the production and assembly of a functioning T3SS and reducing the swimming and swarming ability of DC300 due to the loss of flagella [95]. However, the study also showed that DC3000 has developed efflux pumps like MexAB-OprM that prevent the intracellular build-up of flavonoids. Thanks to these efflux pumps further colonisation by the bacteria is promoted by the subsequent secretion of T3 effector (T3E) into plant cells thereby inhibiting continued generation of these antimicrobials. The identified C-glycosylated flavones in oat are also found in other cereals like wheat, rice and maize. Vitexin, isovitexin, orientin and isoorientin are among the most commonly found C-glycosides and are derived from naringenin as flavanone precursor (Fig 10). Known derivatives of vitexin include isovitexin, vitexin-2-O-rhamnoside, methylvitexin, rhamnopyranosyl‐ and vitexin‐2‐O‐xyloside [96,97]. These compounds are known as radical scavengers and antioxidants, while the antimicrobial activity of apigenin, luteolin, and their C-glucosides were tested and found to generally be more potent against Gram-negative bacteria than Gram-positive ones [98].

Among the triggered alterations, linoleic—and linolenic acid fatty acids were identified. Previous metabolomic studies have highlighted the presence of oxygenated lipids in plant defence in response to biotic stress [99]. These fatty acids are responsible for the biosynthesis of JA and derivatives *via* the octadecanoic pathway. Jasmonates are signalling hormones produced in response to pathogen attack and causes the plant to adjust its metabolism to produce potent defensive secondary metabolites like phenolics, flavonoids, alkaloids and terpenes [100]. In this study, ethyl 7-epi-12-hydroxyjasmonate glucoside was identified among the discriminatory metabolites in the treated groups of *Ps-c* and DC3000. This metabolite has been linked to the JA metabolic pathway, and its production was found to be induced when plants detect a microorganism [101].

Alkaloid metabolites are essential for plant defence, particularly as antibacterial substances. A range of amino acids namely aspartate, lysine, tyrosine, and tryptophan (identified as discriminatory, Table 1) are precursors in the synthesis of alkaloids [102]. Due to the presence of nitrogen atoms (proton accepting) and amine hydrogen group(s) (proton donating), these compounds exhibit outstanding biological activities that are mostly attributed to their capacity to form hydrogen bonds with proteins, enzymes, and receptors [103]. Two alkaloids were identified as discriminatory in the treated groups namely hordenine and feruloylserotonin that possibly contributed to the antimicrobial activity against the respective pathovars. Hordenine was only identified as discriminatory for the *Ps-c* and *Ps-t* treated groups. Hordenine is a phenethylamine alkaloid with numerous bioactivities, including antibiotic action against microbes, that was first discovered in barley and is now also identified in a variety of grasses. Hordenine was reported to act as a plant allelochemical that can restrict the growth of weeds or guard against pathogen attacks. Additionally, it has also been found to be involved in plant defence responses through the activation of JA-dependent pathways [104]. Feruloylserotonin is the other alkaloid (also a hydroxycinnamic acid amide) that has been identified in *Ps-t* treated groups. Relatedly, a study on rice showed that the tryptophan pathway is activated after treatment with *Bipolaris oryzae*, producing serotonin and its amide-conjugates [105]. Moreover, it was reported that serotonin is absorbed into the cell walls of the rice plant tissues and that mutants lacking the elements required for serotonin production and deposition shows an increased susceptibility towards pathogen infection.

## Conclusions and future perspectives

In order to improve on defence strategies against pathogens that are constantly evolving, the development of a systems biology approach to elucidate the biochemical and molecular mechanisms underlying plant immune responses has become essential. Oat plants have received very little attention, with minimal studies on how these plants respond at a metabolic level to pathogenic threats. The adaptive metabolic alterations and mechanisms involved in the oat response to pathogen infection with a range of *P*. *syringe* pathovars have therefore been covered in this work.

The untargeted metabolomics approach was successfully implemented to acquire a deeper understanding of how oat plants defend themselves at a metabolome level against biotic stress. A broad-based chemical defence response was revealed by multivariate data analysis, which also highlighted signature metabolites and discriminatory markers from a range of metabolite classes. The metabolic markers identified in this study show variations in activated oat defences and shed light on the processes and interactions that underlie this plant pathosystem. The study also revealed metabolic pathways that might be responsible for the metabolic changes brought on by inoculation. The observed trend in the metabolite profiles at various time points provided reasonable support that the respective treatments caused defence activation involving the linoleic acid pathway and secondary metabolite biosynthesis pathways. Phenylalanine, tyrosine, and tryptophan metabolism, the phenylpropanoid pathway and the isoquinoline alkaloid biosynthesis pathway, were highlighted as associated with the synthesis of metabolites that belong to the fatty acid, amino acid, phenolic acid, phenolic amide, saponin, flavonoid, and alkaloid classes. Combined, the different biological functions of the secondary metabolites are essential in providing protection against pathogen infections and maintaining it under a variety of environmental conditions.

In terms of nonhost resistance where the plant is able to restrict the pathogen from causing severe infection due to the pathogen not being well adapted in terms of effectors to overthrow plant defences, it can be seen that the Dunnart cultivar showed great overlapping responses to inoculation with the nonhost pathogens (*Ps-t*, DC3000 and *hrcC*^*−*^). Overlapping responses can also be noted among the host and nonhost treated plants as can be seen with the activation of similar metabolic pathways. This might be the result of converging signalling pathways but also the relative size of the precursor flux through contributing pathways as well as the timing and extent of the initiating elicitation events. However, some metabolites were found to be present in the host response (*Ps-c*) and absent in the nonhost response (*Ps-t*) (e.g. avenanthramide A, B and O/R) and *vice versa* in *Ps-t* but not *Ps-c* (e.g. feruloylserotonin).

By comparing the responses of the *hrcC*^*−*^ treated group *vs* DC3000, it is noted that even though the mutant did not cause visual symptoms, PTI was sufficient to initiate many of the same responses as the host response to *Ps-c* where great overlap was seen between these treated groups. This suggests that the pathways involved in the synthesis of key defence metabolites (like avenanthramides) are induced as an early response to initial PAMP recognition, even in the absence of subsequent injection of effectors. The greatest difference between the *hrcC*^*−*^ and DC3000 treated groups is seen in the presence of phenolics and flavonoids with the *hrcC*^*−*^ treatment showing a greater abundance of these metabolite classes compared to that of DC3000. This could be due to the DC3000 secreting effectors into the plant that are able to reduce the mobilisation of small molecule defences beyond the initial PTI response.

Understanding what prevents DC3000 and the *hrcC* mutant from being virulent in oat plants and other non-host plants could be beneficial for future research. Although oat is an economically important food crop, limited in-depth research is currently being conducted. Thus, a disease model including the DC3000 and *hrcC* mutants’ effect on oat would be a valuable reference for crop breeding and resistance studies. This could provide a greater understanding of how the host manages infection by *P*. *syringae* pathovars and could lead to insight into whether effector virulence is target specific in different plants, requiring the use of specific effectors for different plants within a strain’s host range. Disease resistance and the study thereof is crucial for crop defense, and more so the study of non-host resistance since it is considered to be more durable and outlast host-specific resistance.

The data presented in this study could also add to growing databases like the SCIPDb [6] where the molecular and phenotypical response of plants to a combination of stressors are highlighted. Climate change may increase disease risks by altering pathogen evolution and facilitating pathogen spread to new areas. This type of research will therefore become increasingly important when it comes to defining ‘what it takes to be a pathogen’ [15] and as plant disease outbreaks continue to threaten global food security. This study will ultimately provide a basis for insights into the dynamics of the oat metabolome under diverse biotic stresses which, in turn, can be applied in future studies to improve crop resistance and contribute to the development of new strategies for crop improvement endeavours.

## Supporting information

S1 Fig *Pseudomonas syringae* interaction with the plant cell and the respective PAMPs commonly recognised by plant cell receptors. (DOCX)

S2 Fig The use of mass spectral fragmentation data for the confirmation of elemental composition and potential structural elucidation. (DOCX)

S1 Table Statistical validation of the generated OPLS-DA models from the ESI(–) data of oat seedlings treated with the respective *Pseudomonas syringae* pathovars (*Ps-c*, *Ps-t*, DC3000 and *hrcC* mutant). (DOCX)

## References

[1] Gimenez E, Salinas M, Manzano-Agugliaro F. Worldwide research on plant defense against biotic stresses as improvement for sustainable agriculture. Sustainability. 2018; 10:391.

[2] Gull A, Lone AA, Wani NU. Biotic and abiotic stresses in plants. Abiotic and biotic stress in plants. 2019 (pp.1–19). InTechOpen, London, U.K.

[3] González-Lamothe R, Mitchell G, Gattuso M, Diarra MS, Malouin F, Bouarab K. Plant antimicrobial agents and their effects on plant and human pathogens. International Journal of Molecular Sciences. 2009; 10:3400–3419. doi: 10.3390/ijms10083400 20111686 PMC2812829

[4] Moore JW, Loake GJ, Spoel SH. Transcription dynamics in plant immunity. The Plant Cell. 2011; 23:2809–20. doi: 10.1105/tpc.111.087346 21841124 PMC3180793

[5] Wu S, Chen W, Lu S, Zhang H, Yin L. Metabolic engineering of shikimic acid biosynthesis pathway for the production of shikimic acid and its branched products in microorganisms: advances and prospects. Molecules. 2022; 27:4779. doi: 10.3390/molecules27154779 35897952 PMC9332510

[6] Priya P, Patil M, Pandey P, Singh A, Babu VS, Senthil‐Kumar M. Stress combinations and their interactions in plants database: a one‐stop resource on combined stress responses in plants. The Plant Journal. 2023; 116:1097–1117. doi: 10.1111/tpj.16497 37824297

[7] Senthil-Kumar M, Mysore KS. Nonhost resistance against bacterial pathogens: retrospectives and prospects. Annual Review of Phytopathology. 2013; 51:407–427. doi: 10.1146/annurev-phyto-082712-102319 23725473

[8] Underwood W. The plant cell wall: a dynamic barrier against pathogen invasion. Frontiers in Plant Science. 2012; 3:85. doi: 10.3389/fpls.2012.00085 22639669 PMC3355688

[9] Andersen EJ, Ali S, Byamukama E, Yen Y, Nepal MP. Disease resistance mechanisms in plants. Genes. 2018; 9:339. doi: 10.3390/genes9070339 29973557 PMC6071103

[10] Kaur S, Samota MK, Choudhary M, Choudhary M, Pandey AK, Sharma A, et al. How do plants defend themselves against pathogens-Biochemical mechanisms and genetic interventions. Physiology and Molecular Biology of Plants. 2022; 28:485–504. doi: 10.1007/s12298-022-01146-y 35400890 PMC8943088

[11] Jones JD, Dangl JL. The plant immune system. Nature. 2006; 444:323–329. doi: 10.1038/nature05286 17108957

[12] Asai S, Shirasu K. Plant cells under siege: plant immune system versus pathogen effectors. Current Opinion in Plant Biology. 2015; 28:1–8. doi: 10.1016/j.pbi.2015.08.008 26343014

[13] Hajam IA, Dar PA, Shahnawaz I, Jaume JC, Lee JH. Bacterial flagellin—a potent immunomodulatory agent. Experimental and Molecular Medicine. 2017; 49:373. doi: 10.1038/emm.2017.172 28860663 PMC5628280

[14] Tang D, Wang G, Zhou JM. Receptor kinases in plant-pathogen interactions: more than pattern recognition. The Plant Cell. 2017; 29:618–637. doi: 10.1105/tpc.16.00891 28302675 PMC5435430

[15] Xin XF, Kvitko B, He SY. *Pseudomonas syringae*: what it takes to be a pathogen. Nature Reviews Microbiology. 2018; 16:316–328. 29479077 10.1038/nrmicro.2018.17 PMC5972017

[16] Horbach R, Navarro-Quesada AR, Knogge W, Deising HB. When and how to kill a plant cell: infection strategies of plant pathogenic fungi. Journal of Plant Physiology. 2011; 168:51–62. doi: 10.1016/j.jplph.2010.06.014 20674079

[17] Korotkov KV, Sandkvist M, Hol WG. The type II secretion system: biogenesis, molecular architecture and mechanism. Nature Reviews Microbiology. 2012; 10:336–51. doi: 10.1038/nrmicro2762 22466878 PMC3705712

[18] Bender CL, Alarcón-Chaidez F, Gross DC. Pseudomonas syringae phytotoxins: mode of action, regulation, and biosynthesis by peptide and polyketide synthetases. Microbiology and Molecular Biology Reviews. 1999; 63:266–292. doi: 10.1128/MMBR.63.2.266-292.1999 10357851 PMC98966

[19] Zheng XY, Spivey NW, Zeng W, Liu PP, Fu ZQ, Klessig DF, et al. Coronatine promotes *Pseudomonas syringae* virulence in plants by activating a signaling cascade that inhibits salicylic acid accumulation. Cell Host and Microbe. 2012; 11:587–96. 22704619 10.1016/j.chom.2012.04.014 PMC3404825

[20] Balint‐Kurti P. The plant hypersensitive response: concepts, control and consequences. Molecular Plant Pathology. 2019; 20:1163–1178. doi: 10.1111/mpp.12821 31305008 PMC6640183

[21] van der Hoorn RA, Kamoun S. From guard to decoy: a new model for perception of plant pathogen effectors. The Plant Cell. 2008; 20:2009–2017. doi: 10.1105/tpc.108.060194 18723576 PMC2553620

[22] Cesari S. Multiple strategies for pathogen perception by plant immune receptors. New Phytologist. 2018; 219:17–24. doi: 10.1111/nph.14877 29131341

[23] Niks RE, Marcel TC. Nonhost and basal resistance: how to explain specificity?. New Phytologist. 2009; 182:817–28. doi: 10.1111/j.1469-8137.2009.02849.x 19646067

[24] Gill US, Lee S, Mysore KS. Host versus nonhost resistance: distinct wars with similar arsenals. Phytopathology. 2015; 105:580–587. doi: 10.1094/PHYTO-11-14-0298-RVW 25626072

[25] Ayliffe M, Sørensen CK. Plant nonhost resistance: paradigms and new environments. Current Opinion in Plant Biology. 2019; 50:104–113. doi: 10.1016/j.pbi.2019.03.011 31075541

[26] Mysore KS, Ryu CM. Nonhost resistance: how much do we know?. Trends in Plant Science. 2004; 9:97–104. doi: 10.1016/j.tplants.2003.12.005 15102376

[27] Ishiga Y, Ishiga T, Ikeda Y, Matsuura T, Mysore KS. NADPH-dependent thioredoxin reductase C plays a role in nonhost disease resistance against *Pseudomonas syringae* pathogens by regulating chloroplast-generated reactive oxygen species. PeerJ. 2016; 4:1938. 10.7717/peerj.1938 PMC4860297 27168965

[28] Wittstock U, Burow M. Glucosinolate breakdown in Arabidopsis: mechanism, regulation and biological significance. The Arabidopsis book/American Society of Plant Biologists. 2010; 8:e0134. doi: 10.1199/tab.0134 22303260 PMC3244901

[29] Oh S, Choi D. Receptor-mediated nonhost resistance in plants. Essays in Biochemistry. 2022; 66:435–445. doi: 10.1042/EBC20210080 35388900 PMC9528085

[30] Castro-Moretti FR, Gentzel IN, Mackey D, Alonso AP. Metabolomics as an emerging tool for the study of plant–pathogen interactions. Metabolites. 2020; 10:52. doi: 10.3390/metabo10020052 32013104 PMC7074241

[31] Tugizimana F, Piater L, Dubery I. Plant metabolomics: A new frontier in phytochemical analysis. South African Journal of Science. 2013; 109:1–11.

[32] Johnson CH, Ivanisevic J, Siuzdak G. Metabolomics: beyond biomarkers and towards mechanisms. Nature Reviews Molecular Cell Biology. 2016; 17:451–459. doi: 10.1038/nrm.2016.25 26979502 PMC5729912

[33] Isah T. Stress and defense responses in plant secondary metabolites production. Biological Research. 2019; 52:39. doi: 10.1186/s40659-019-0246-3 31358053 PMC6661828

[34] Salam U, Ullah S, Tang ZH, Elateeq AA, Khan Y, Khan J, et al. Plant metabolomics: an overview of the role of primary and secondary metabolites against different environmental stress factors. Life. 2023; 13:706. doi: 10.3390/life13030706 36983860 PMC10051737

[35] Serag A, Salem MA, Gong S, Wu JL, Farag MA. Decoding metabolic reprogramming in plants under pathogen attacks, a comprehensive review of emerging metabolomics technologies to maximize their applications. Metabolites. 2023; 13:424. doi: 10.3390/metabo13030424 36984864 PMC10055942

[36] Elliott C. Halo-blight of oats. Journal of Agricultural Research. 1920; XIX:139–172.

[37] Persson P, Sletten A. Halo blight of oats in Scandinavia. In: *Proceedings of the 10th International Conference on Plant Pathogenic Bacteria*, Charlottetown, Prince Edward Island, Canada, 2001; Dordrecht: Springer Netherlands. pp. 265–268.

[38] Kim YC. First report of oat halo blight caused by *Pseudomonas coronafaciens* in South Korea. Plant Disease. 2020;10:1853.

[39] Pretorius CJ, Dubery IA. Integration of targeted metabolome and transcript profiling of *Pseudomonas syringae*-triggered changes in defence-related phytochemicals in oat plants. Planta. 2024; 260:8. 38789631 10.1007/s00425-024-04435-w PMC11126498

[40] Gorash A, Armonienė R, Mitchell Fetch J, Liatukas Ž, Danytė V. Aspects in oat breeding: nutrition quality, nakedness and disease resistance, challenges and perspectives. Annals of Applied Biology. 2017; 171:281–302.

[41] Kumar R, Bohra A, Pandey AK, Pandey MK, Kumar A. Metabolomics for plant improvement: status and prospects. Frontiers in Plant Science. 2017; 8:1302. doi: 10.3389/fpls.2017.01302 28824660 PMC5545584

[42] Hiruma K, Saijo Y. Plant inoculation with the fungal leaf pathogen *Colletotrichum higginsianum*. Environmental Responses in Plants: Methods and Protocols. 2016; 313–318. doi: 10.1007/978-1-4939-3356-3_24 26867633

[43] Felix G, Duran JD, Volko S, Boller T. Plants have a sensitive perception system for the most conserved domain of bacterial flagellin. The Plant Journal. 1999; 18:265–276. doi: 10.1046/j.1365-313x.1999.00265.x 10377992

[44] Zeiss DR, Steenkamp PA, Piater LA, Dubery IA. Altered metabolomic states elicited by Flg22 and FlgII-28 in *Solanum lycopersicum*: intracellular perturbations and metabolite defenses. BMC Plant Biology. 202; 21:1–7. 10.1186/s12870-021-03200-5 PMC8456652 34548030

[45] Mott GA, Desveaux D, Guttman DS. A high-sensitivity, microtiter-based plate assay for plant pattern-triggered immunity. Molecular Plant-Microbe Interactions. 2018; 31:499–504. doi: 10.1094/MPMI-11-17-0279-TA 29199888

[46] Broadhurst D, Goodacre R, Reinke SN, Kuligowski J, Wilson ID, Lewis MR, et al. Guidelines and considerations for the use of system suitability and quality control samples in mass spectrometry assays applied in untargeted clinical metabolomic studies. Metabolomics. 2018; 14:1–7. 29805336 10.1007/s11306-018-1367-3 PMC5960010

[47] Tugizimana F, Steenkamp PA, Piater LA, Dubery IA. Multi-platform metabolomic analyses of ergosterol-induced dynamic changes in *Nicotiana tabacum* cells. PloS one. 2014; 9:e87846. doi: 10.1371/journal.pone.0087846 24498209 PMC3909234

[48] Zeiss DR, Mhlongo MI, Tugizimana F, Steenkamp PA, Dubery IA. Comparative metabolic phenotyping of tomato (*Solanum lycopersicum*) for the identification of metabolic signatures in cultivars differing in resistance to *Ralstonia solanacearum*. International Journal of Molecular Sciences. 2018; 19:2558. doi: 10.3390/ijms19092558 30158424 PMC6163672

[49] Trygg J, Holmes E, Lundstedt T. Chemometrics in metabonomics. Journal of Proteome Research. 2007; 6:469–479. doi: 10.1021/pr060594q 17269704

[50] Worley B, Powers R. Multivariate analysis in metabolomics. Current Metabolomics. 2013; 1:92–107. doi: 10.2174/2213235X11301010092 26078916 PMC4465187

[51] Salek RM, Steinbeck C, Viant MR, Goodacre R, Dunn WB. The role of reporting standards for metabolite annotation and identification in metabolomic studies. Gigascience. 2013; 2:2047–17X. doi: 10.1186/2047-217X-2-13 24131531 PMC3853013

[52] Pang Z, Chong J, Zhou G, de Lima Morais DA, Chang L, Barrette M, et al. MetaboAnalyst 5.0: Narrowing the gap between raw spectra and functional insights. Nucleic Acids Research. 2021; 49:W388–W396. doi: 10.1093/nar/gkab382 34019663 PMC8265181

[53] Peñaloza-Vázquez A, Preston GM, Collmer A, Bender CL. Regulatory interactions between the Hrp type III protein secretion system and coronatine biosynthesis in *Pseudomonas syringae* pv. *tomato* DC3000. Microbiology. 2000; 146:2447–56. 11021921 10.1099/00221287-146-10-2447

[54] Eriksson L, Trygg J, Wold S. CV‐ANOVA for significance testing of PLS and OPLS® models. Journal of Chemometrics. 2008; 22:594–600.

[55] Xia J, Wishart DS. Metabolomic data processing, analysis, and interpretation using MetaboAnalyst. Current Protocols in Bioinformatics. 2011; 34:1–14. doi: 10.1002/0471250953.bi1410s34 21633943

[56] Dong NQ, Lin HX. Contribution of phenylpropanoid metabolism to plant development and plant–environment interactions. Journal of Integrative Plant Biology. 2021; 63:180–209. doi: 10.1111/jipb.13054 33325112

[57] Mathesius U. Flavonoid functions in plants and their interactions with other organisms. Plants. 2018; 7:30. doi: 10.3390/plants7020030 29614017 PMC6027123

[58] Pratyusha S. Phenolic compounds in the plant development and defense: an overview. Plant Stress Physiology-Perspectives in Agriculture. 2022; 125–140.

[59] Mandal SM, Chakraborty D, Dey S. Phenolic acids act as signaling molecules in plant-microbe symbioses. Plant Signaling and Behavior. 2010; 5:359–368. doi: 10.4161/psb.5.4.10871 20400851 PMC2958585

[60] Pretorius CJ, Dubery IA. Avenanthramides, Distinctive Hydroxycinnamoyl Conjugates of Oat, *Avena sativa* L.: An Update on the Biosynthesis, Chemistry, and Bioactivities. Plants. 2023; 12:1388. 36987077 10.3390/plants12061388 PMC10055937

[61] He M, Ding NZ. Plant unsaturated fatty acids: multiple roles in stress response. Frontiers in Plant Science. 2020; 11:562785. doi: 10.3389/fpls.2020.562785 33013981 PMC7500430

[62] Zhang L, Zhang F, Melotto M, Yao J, He SY. Jasmonate signaling and manipulation by pathogens and insects. Journal of Experimental Botany. 2017; 68:1371–1385. doi: 10.1093/jxb/erw478 28069779 PMC6075518

[63] Naveed ZA, Wei X, Chen J, Mubeen H, Ali GS. The PTI to ETI continuum in Phytophthora-plant interactions. Frontiers in Plant Science. 2020; 11:593905. doi: 10.3389/fpls.2020.593905 33391306 PMC7773600

[64] Anderson JP, Gleason CA, Foley RC, Thrall PH, Burdon JB, Singh KB. Plants versus pathogens: an evolutionary arms race. Functional Plant Biology. 2010; 37:499–512. doi: 10.1071/FP09304 21743794 PMC3131095

[65] Dubey N. and Singh K. Role of NBS-LRR proteins in plant defense. In Molecular Aspects of Plant-Pathogen Interaction, 2018 (pp. 115–138). Springer, Heidelberg, Germany.

[66] Panstruga R, Moscou MJ. What is the molecular basis of nonhost resistance?. Molecular Plant-Microbe Interactions. 2020; 33:1253–1264. doi: 10.1094/MPMI-06-20-0161-CR 32808862

[67] Saini P, Gani M, Saini P, Bhat JA, Francies RM, Negi N, et al. Molecular breeding for resistance to economically important diseases of fodder oat. In Disease Resistance in Crop Plants: Molecular, Genetic and Genomic Perspectives, Wani S., (ed.). 2019 (pp. 199–239). Springer, Cham, Switzerland.

[68] Riley MB, Williamson MR, Maloy O. Plant disease diagnosis. The Plant Health Instructor. 2016; 10.

[69] Tugizimana F, Mhlongo MI, Piater LA, Dubery IA. Metabolomics in plant priming research: the way forward?. International Journal of Molecular Sciences. 2018; 19:1759. doi: 10.3390/ijms19061759 29899301 PMC6032392

[70] Preston GM. Profiling the extended phenotype of plant pathogens: challenges in bacterial molecular plant pathology. Molecular Plant Pathology. 2017; 18:443–456. doi: 10.1111/mpp.12530 28026146 PMC6638297

[71] Hafez YM, Mourad RY, Nasr EB, Attia KO, Abdelaal KA, Ghazy AI, et al. Biochemical and molecular characterization of non-host resistance keys in food crops. Saudi Journal of Biological Sciences. 2020; 27:1091–1099. doi: 10.1016/j.sjbs.2019.12.041 32256170 PMC7105668

[72] Yoda H, Fujimura K, Takahashi H, Munemura I, Uchimiya H, Sano H. Polyamines as a common source of hydrogen peroxide in host-and nonhost hypersensitive response during pathogen infection. Plant Molecular Biology. 2009; 70:103–112. doi: 10.1007/s11103-009-9459-0 19190986

[73] Torres MA, Jones JD, Dangl JL. Reactive oxygen species signaling in response to pathogens. Plant Physiology. 2006; 141:373–378. doi: 10.1104/pp.106.079467 16760490 PMC1475467

[74] Zurbriggen MD, Carrillo N, Hajirezaei MR. ROS signaling in the hypersensitive response: when, where and what for?. Plant Signaling and Behavior. 2010; 5:393–396. doi: 10.4161/psb.5.4.10793 20383072 PMC2958590

[75] Smith JM, Heese A. Rapid bioassay to measure early reactive oxygen species production in Arabidopsis leave tissue in response to living *Pseudomonas syringae*. Plant Methods. 2014; 10:1–9. 24571722 10.1186/1746-4811-10-6 PMC3941562

[76] Almagro L, Gómez Ros LV, Belchi-Navarro S, Bru R, Ros Barceló A, Pedreño MA. Class III peroxidases in plant defence reactions. Journal of Experimental Botany. 2009; 60:377–390. doi: 10.1093/jxb/ern277 19073963

[77] Thakker JN, Patel S, Dhandhukia PC. Induction of defense‐related enzymes in banana plants: effect of live and dead pathogenic strain of *Fusarium oxysporum* f. sp. *cubense*. International Scholarly Research Notices. 2013; 2013:1–6. 10.5402/2013/601303 PMC4403610 25969777

[78] Xu X, Chen Y, Li B, Zhang Z, Qin G, Chen T, et al. Molecular mechanisms underlying multi-level defense responses of horticultural crops to fungal pathogens. Horticulture Research. 2022; 9:066. doi: 10.1093/hr/uhac066 35591926 PMC9113409

[79] Tao Y, Xie Z, Chen W, Glazebrook J, Chang HS, Han B, et al. Quantitative nature of Arabidopsis responses during compatible and incompatible interactions with the bacterial pathogen *Pseudomonas syringae*. The Plant Cell. 2003; 15:317–330. 12566575 10.1105/tpc.007591 PMC141204

[80] Roumani M, Duval RE, Ropars A, Risler A, Robin C, Larbat R. Phenolamides: Plant specialized metabolites with a wide range of promising pharmacological and health-promoting interests. Biomedicine and Pharmacotherapy. 2020; 131:110762. doi: 10.1016/j.biopha.2020.110762 33152925

[81] Zeiss DR, Piater LA, Dubery IA. Hydroxycinnamate amides: Intriguing conjugates of plant protective metabolites. Trends in Plant Science. 2021; 26:184–195. doi: 10.1016/j.tplants.2020.09.011 33036915

[82] Matsukawa T, Ishihara A, Iwamura H. Induction of anthranilate synthase activity by elicitors in oats. Zeitschrift für Naturforschung C. 2002; 57:121–128. doi: 10.1515/znc-2002-1-221 11926523

[83] Yang Q, Xuan Trinh H, Imai S, Ishihara A, Zhang L, Nakayashiki H, et al. Analysis of the involvement of hydroxyanthranilate hydroxycinnamoyltransferase and caffeoyl-CoA 3-O-methyltransferase in phytoalexin biosynthesis in oat. Molecular Plant-Microbe Interactions. 2004; 17:81–89. doi: 10.1094/MPMI.2004.17.1.81 14714871

[84] Li Z, Chen Y, Meesapyodsuk D, Qiu X. The biosynthetic pathway of major avenanthramides in oat. Metabolites. 2019; 9:163. doi: 10.3390/metabo9080163 31394723 PMC6724135

[85] Collins FW, Mullin WJ. High-performance liquid chromatographic determination of avenanthramides, N-aroylanthranilic acid alkaloids from oats. Journal of Chromatography A. 1988; 445:363–370.

[86] Taofiq O, González-Paramás AM, Barreiro MF, Ferreira IC. Hydroxycinnamic acids and their derivatives: Cosmeceutical significance, challenges and future perspectives, a review. Molecules. 2017; 22:281. doi: 10.3390/molecules22020281 28208818 PMC6155946

[87] Shahidi F, Chandrasekara A. Hydroxycinnamates and their in vitro and in vivo antioxidant activities. Phytochemistry Reviews. 2010; 9:147–170.

[88] Ishihara A, Kojima K, Fujita T, Yamamoto Y, Nakajima H. New series of avenanthramides in oat seed. Bioscience, Biotechnology, and Biochemistry. 2014; 78:1975–1983. doi: 10.1080/09168451.2014.946390 25117953

[89] Morrissey JP, Osbourn AE. Fungal resistance to plant antibiotics as a mechanism of pathogenesis. Microbiology and Molecular Biology Reviews. 1999; 63:708–724. doi: 10.1128/MMBR.63.3.708-724.1999 10477313 PMC103751

[90] Mugford ST, Osbourn A. Saponin synthesis and function. Isoprenoid synthesis in plants and microorganisms: New concepts and experimental approaches. 2013 (pp. 405–424) Springer. New York. NY.

[91] Arabski M, Węgierek-Ciuk A, Czerwonka G, Lankoff A, Kaca W. Effects of saponins against clinical E. coli strains and eukaryotic cell line. BioMed Research International. 2012; 2012:286216. 10.1155/2012/286216 PMC3303633 22500084

[92] Sun W, Shahrajabian MH. Therapeutic potential of phenolic compounds in medicinal plants—Natural health products for human health. Molecules. 2023; 28:1845. doi: 10.3390/molecules28041845 36838831 PMC9960276

[93] Santos-Sánchez NF, Salas-Coronado R, Hernández-Carlos B, Villanueva-Cañongo C. Shikimic acid pathway in biosynthesis of phenolic compounds. In: Plant Physiological Aspects of Phenolic Compounds, Soto-Hernández M, García-Mateos R, Palma-Tenango M, eds., InTechOpen, London, UK, 2019; pp. 1–15.

[94] Dehghanian Z, Habibi K, Dehghanian M, Aliyar S, Lajayer BA, Astatkie T, et al. Reinforcing the bulwark: Unravelling the efficient applications of plant phenolics and tannins against environmental stresses. Heliyon. 2022; 8. doi: 10.1016/j.heliyon.2022.e09094 35309390 PMC8927939

[95] Vargas P, Farias GA, Nogales J, Prada H, Carvajal V, Barón M, et al. Plant flavonoids target *Pseudomonas syringae* pv. *tomato* DC 3000 flagella and type III secretion system. Environmental Microbiology Reports. 2013; 5:841–850. 24249293 10.1111/1758-2229.12086

[96] Vanegas KG, Larsen AB, Eichenberger M, Fischer D, Mortensen UH, Naesby M. Indirect and direct routes to C-glycosylated flavones in *Saccharomyces cerevisiae*. Microbial Cell Factories. 2018; 1–10. 29986709 10.1186/s12934-018-0952-5 PMC6036675

[97] Babaei F, Moafizad A, Darvishvand Z, Mirzababaei M, Hosseinzadeh H, Nassiri‐Asl M. Review of the effects of vitexin in oxidative stress‐related diseases. Food Science and Nutrition. 2020; 8:2569–2580. doi: 10.1002/fsn3.1567 32566174 PMC7300089

[98] Karpiński T, Adamczak A, and Ożarowski M. November. Antibacterial activity of apigenin, luteolin, and their C-glucosides. 2019 (pp.1–9). In Proceedings of the 5th International Electronic Conference on Medicinal Chemistry. Poland.

[99] Pretorius CJ, Zeiss DR, Dubery IA. The presence of oxygenated lipids in plant defense in response to biotic stress: A metabolomics appraisal. Plant Signaling and Behavior. 2021; 16:1989215. doi: 10.1080/15592324.2021.1989215 34968410 PMC9208797

[100] Wasternack C, Strnad M. Jasmonates: News on occurrence, biosynthesis, metabolism and action of an ancient group of signaling compounds. International Journal of Molecular Sciences. 2018; 19:2539. doi: 10.3390/ijms19092539 30150593 PMC6164985

[101] Mayo-Prieto S, Marra R, Vinale F, Rodríguez-González Á, Woo SL, Lorito M, et al. Effect of *Trichoderma velutinum* and *Rhizoctonia solani* on the metabolome of bean plants (*Phaseolus vulgaris* L.). International Journal of Molecular Sciences. 2019; 20:549. doi: 10.3390/ijms20030549 30696057 PMC6387467

[102] Parthasarathy A, Borrego EJ, Savka MA, Dobson RC, Hudson AO. Amino acid–derived defense metabolites from plants: A potential source to facilitate novel antimicrobial development. Journal of Biological Chemistry. 2021; 296. 10.1016/j.jbc.2021.100438 PMC8024917 33610552

[103] Thawabteh A, Juma S, Bader M, Karaman D, Scrano L, Bufo SA, et al. The biological activity of natural alkaloids against herbivores, cancerous cells and pathogens. Toxins. 2019; 11:656. doi: 10.3390/toxins11110656 31717922 PMC6891610

[104] Ishiai S, Kondo H, Hattori T, Mikami M, Aoki Y, Enoki S, et al. Hordenine is responsible for plant defense response through jasmonate-dependent defense pathway. Physiological and Molecular Plant Pathology. 2016; 96:94–100.

[105] Ishihara A, Hashimoto Y, Tanaka C, Dubouzet JG, Nakao T, Matsuda F, et al. The tryptophan pathway is involved in the defense responses of rice against pathogenic infection via serotonin production. The Plant Journal. 2008; 54:481–495. doi: 10.1111/j.1365-313X.2008.03441.x 18266919

