# Supplementary material for: Metabolome profiling dissects the oat (Avena sativa L.) innate immune response to Pseudomonas syringae pathovars
Source: PLoS One. 2025 Feb 3;20(2):e0311226. doi: 10.1371/journal.pone.0311226 (PMC11790117; doi:10.1371/journal.pone.0311226)
Supplement: S1 Fig — (DOCX) [file pone.0311226.s001.docx]

**Supporting information - S1 Fig**


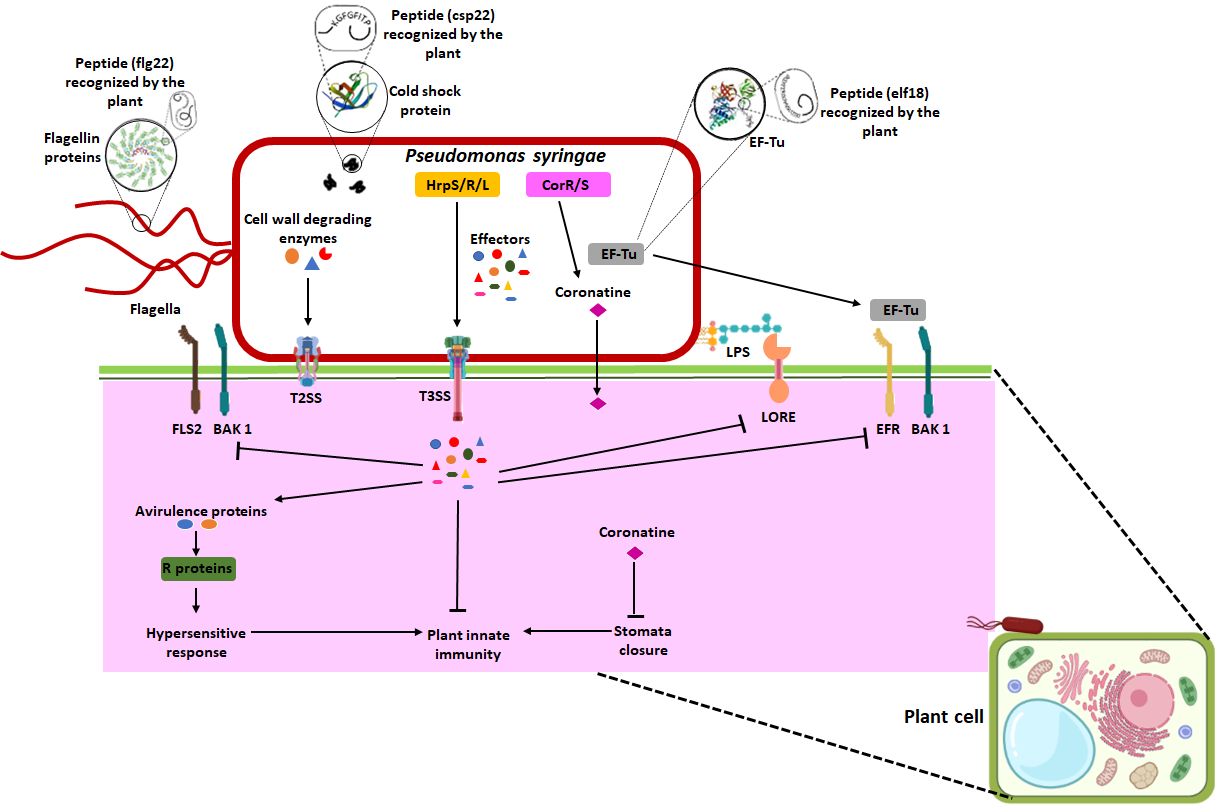


**S1 Fig. *Pseudomonas syringae* interaction with the plant cell and the respective PAMPs commonly recognised by plant cell receptors.** *P. syringae* injects type III effectors (T3Es) into the host cell *via* the T3 secretion system (T3SS). PAMPs from *P. syringae* are detected *via* receptor-like kinases (RLKs) such as FLS2, resulting in pattern-triggered immunity (PTI). T3Es inhibit PTI by targeting signalling events resulting from PAMP receptor complexes (in this case, the pattern recognition receptor FLS2 and its co-receptor BAK1). The recognition of effector proteins initiates effector-triggered immunity (ETI) and produces a hypersensitive response (HR).
