# Supplementary material for: Metabolome profiling dissects the oat (Avena sativa L.) innate immune response to Pseudomonas syringae pathovars
Source: PLoS One. 2025 Feb 3;20(2):e0311226. doi: 10.1371/journal.pone.0311226 (PMC11790117; doi:10.1371/journal.pone.0311226)
Supplement: S2 Fig — (DOCX) [file pone.0311226.s002.docx]

**Supporting information - S2 Fig**


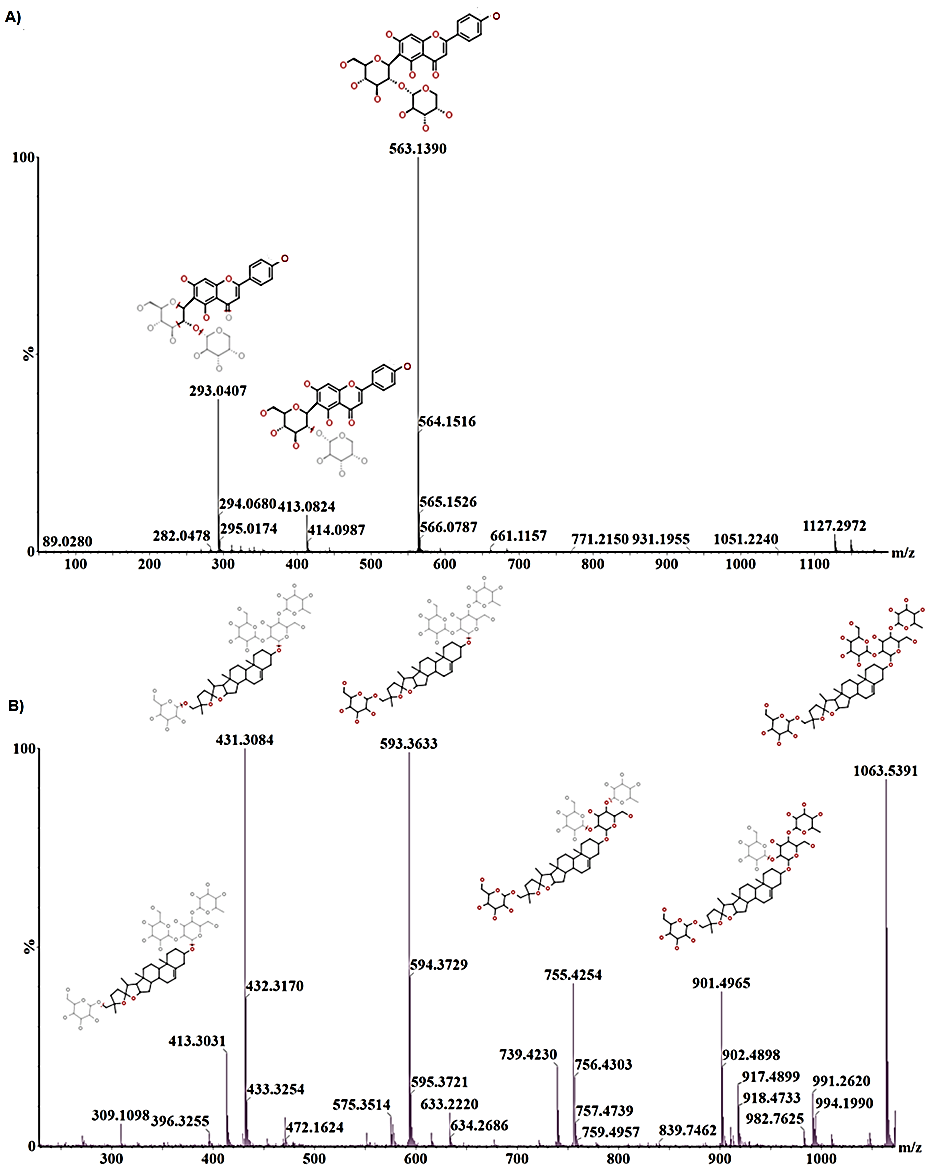


**S2 Fig. The use of mass spectral fragmentation data for the confirmation of elemental composition and potential structural elucidation.** Isovitexin 2''-O-arabinoside **(A)** with the parent ion at *m/z* 563 and two diagnostic fragment ions at *m/z* 413 and 293, representing the utilisation of fragmentation patterns for metabolite annotation. **(B)** Avenacoside A at *m/z* 1063 displaying diagnostic fragments (*m/z* 901, 755, 593, 431, and 413) produced at different collision energies (MS^E^) for structural identification.
