# Supplementary material for: Metabolome profiling dissects the oat (Avena sativa L.) innate immune response to Pseudomonas syringae pathovars
Source: PLoS One. 2025 Feb 3;20(2):e0311226. doi: 10.1371/journal.pone.0311226 (PMC11790117; doi:10.1371/journal.pone.0311226)
Supplement: S1 Table — (DOCX) [file pone.0311226.s003.docx]

**Supporting information - S1 Table**

**S1 Table. Statistical validation of the generated OPLS-DA models from the ESI(–) data of oat seedlings treated with the respective *Pseudomonas syringae* pathovars (*Ps-c, Ps-t,* DC3000 and *hrcC* mutant).** For each of the OPLS-DA models, the estimated R^2^X(cum), R^2^Y(cum), and Q^2^(cum) values are shown. The permutation analysis R^2^ values (n = 100 random permutations) are compared and demonstrated to be much lower than that of the generated OPLS-DA models. The statistical significance of each investigated model was determined using the *p*-value of CV-ANOVA.

| **Model (Day 2)** | **R^2^X(cum)** | **R^2^Y(cum)** | **Q^2^(cum)** | **CV-ANOVA** | **Permutation R^2^** |
| --- | --- | --- | --- | --- | --- |
| Dunnart DC3000 *vs* VC | 0.428 | 0.992 | 0.952 | 1.91e^-08^ | 0.746 |
| Dunnart *hrcC−* *vs* VC | 0.420 | 0.999 | 0.952 | 1.29e^-06^ | 0.961 |
| Dunnart *Ps-c* *vs* VC | 0.477 | 0.998 | 0.936 | 5.86e^-06^ | 0.951 |
| Dunnart *Ps-t* *vs* VC | 0.442 | 0.998 | 0.947 | 2.20e^-06^ | 0.960 |
| Dunnart DC3000 *vs* *hrcC−* | 0.383 | 0.992 | 0.961 | 5.12e^-09^ | 0.786 |
| Dunnart DC3000 *vs* *Ps-c* | 0.342 | 0.989 | 0.932 | 1.81e^-07^ | 0.840 |
| Dunnart DC3000 *vs Ps-t* | 0.435 | 0.994 | 0.853 | 0.00048891 | 0.949 |
| Dunnart *hrcC−* *vs Ps-c* | 0.393 | 0.996 | 0.810 | 0.00185562 | 0.957 |
| Dunnart *hrcC−* *vs Ps-t* | 0.431 | 0.999 | 0.949 | 1.73e^-06^ | 0.942 |
| Dunnart *Ps-c vs Ps-t* | 0.330 | 0.988 | 0.922 | 4.57e^-07^ | 0.881 |
| Dunnart HC *vs* VC | 0.381 | 0.995 | 0.852 | 0.00050643 | 0.963 |
| Dunnart HC *vs* DC3000 | 0.451 | 0.996 | 0.976 | 2.19e^-10^ | 0.747 |
| Dunnart HC *vs* *hrcC−* | 0.350 | 0.994 | 0.943 | 5.83e^-08^ | 0.822 |
| Dunnart HC *vs Ps-c* | 0.429 | 0.992 | 0.933 | 1.66e^-07^ | 0.841 |
| Dunnart HC *vs Ps-t* | 0.400 | 0.995 | 0.950 | 2.54e^-08^ | 0.839 |
| **Model (Day 4)** | **R^2^X(cum)** | **R^2^Y** | **Q^2^** | **CV-ANOVA** | **Permutation R^2^** |
| Dunnart DC3000 *vs* VC | 0.584 | 0.993 | 0.976 | 2.00e^-10^ | 0.729 |
| Dunnart *hrcC−* *vs* VC | 0.569 | 0.995 | 0.911 | 3.45e^-05^ | 0.931 |
| Dunnart *Ps-c vs* VC | 0.552 | 0.984 | 0.949 | 2.94e^-08^ | 0.715 |
| Dunnart *Ps-t vs* VC | 0.579 | 0.992 | 0.970 | 8.75e^-10^ | 0.821 |
| Dunnart DC3000 *vs hrcC−* | 0.623 | 0.997 | 0.957 | 6.69e^-07^ | 0.922 |
| Dunnart DC3000 *vs Ps-c* | 0.457 | 0.998 | 0.847 | 0.00061221 | 0.960 |
| Dunnart DC3000 *vs Ps-t* | 0.494 | 0.998 | 0.904 | 5.33e^-05^ | 0.962 |
| Dunnart *hrcC−* *vs Ps-c* | 0.593 | 0.994 | 0.890 | 0.00010786 | 0.964 |
| Dunnart *hrcC−* *vs* *Ps-t* | 0.577 | 0.978 | 0.872 | 1.03e^-05^ | 0.823 |
| Dunnart *Ps-c vs Ps-t* | 0.468 | 0.996 | 0.832 | 0.00098695 | 0.976 |
| Dunnart HC *vs* VC | 0.511 | 0.998 | 0.908 | 4.09e^-05^ | 0.955 |
| Dunnart HC *vs* DC3000 | 0.507 | 0.996 | 0.979 | 9.58e^-11^ | 0.686 |
| Dunnart HC *vs hrcC−* | 0.531 | 0.988 | 0.934 | 1.47e^-07^ | 0.788 |
| Dunnart HC *vs Ps-c* | 0.474 | 0.992 | 0.967 | 1.86e^-09^ | 0.856 |
| Dunnart HC *vs Ps-t* | 0.511 | 0.995 | 0.972 | 6.40e^-10^ | 0.770 |
| **Model (Day 6)** | **R^2^X(cum)** | **R^2^Y** | **Q^2^** | **CV-ANOVA** | **Permutation R^2^** |
| Dunnart DC3000 *vs* VC | 0.583 | 0.993 | 0.976 | 2.08e^-10^ | 0.763 |
| Dunnart *hrcC−* *vs* VC | 0.568 | 0.995 | 0.911 | 3.56e^-05^ | 0.932 |
| Dunnart *Ps-c vs* VC | 0.551 | 0.984 | 0.949 | 2.93e^-08^ | 0.808 |
| Dunnart *Ps-t vs* VC | 0.576 | 0.992 | 0.970 | 9.35e^-10^ | 0.801 |
| Dunnart DC3000 *vs* *hrcC−* | 0.623 | 0.997 | 0.957 | 6.75e^-07^ | 0.915 |
| Dunnart DC3000 *vs Ps-c* | 0.455 | 0.998 | 0.844 | 0.00067181 | 0.964 |
| Dunnart DC3000 *vs Ps-t* | 0.493 | 0.998 | 0.903 | 5.55e^-05^ | 0.946 |
| Dunnart *hrcC−* *vs Ps-c* | 0.592 | 0.994 | 0.890 | 0.00010476 | 0.953 |
| Dunnart *hrcC−* *vs Ps-t* | 0.576 | 0.978 | 0.872 | 1.06e^-05^ | 0.806 |
| Dunnart *Ps-c vs Ps-t* | 0.467 | 0.996 | 0.830 | 0.00104831 | 0.973 |
| Dunnart HC *vs* VC | 0.510 | 0.999 | 0.906 | 4.54e^-05^ | 0.960 |
| Dunnart HC *vs* DC3000 | 0.506 | 0.996 | 0.979 | 1.02e^-10^ | 0.784 |
| Dunnart HC *vs hrcC−* | 0.531 | 0.988 | 0.935 | 1.41e^-07^ | 0.797 |
| Dunnart HC *vs Ps-c* | 0.473 | 0.992 | 0.966 | 1.97e^-09^ | 0.808 |
| Dunnart HC *vs Ps-t* | 0.509 | 0.995 | 0.971 | 7.23e^-10^ | 0.785 |

VC = vehicle control, HC = healthy control.
